# An Epstein-Barr virus protein interaction map reveals NLRP3 inflammasome evasion via MAVS UFMylation

**DOI:** 10.1016/j.molcel.2023.05.018

**Published:** 2023-06-12

**Authors:** Stephanie Pei Tung Yiu, Cassie Zerbe, David Vanderwall, Edward L. Huttlin, Michael P. Weekes, Benjamin E. Gewurz

**Affiliations:** 1 Division of Infectious Diseases, Brigham and Women’s Hospital, 181 Longwood Avenue, Boston, MA 02115, USA; 2 Harvard Graduate Program in Virology, Boston, MA 02115, USA; 3 Center for Integrated Solutions to Infectious Diseases, Broad Institute and Harvard Medical School, Cambridge, MA 02115, USA; 4 Department of Microbiology, Harvard Medical School, Boston, MA 02115, USA; 5 Cambridge Institute for Medical Research, University of Cambridge, Hills Road, Cambridge CB2 0XY, UK; 6 Department of Cell Biology, Harvard Medical School, Boston, MA 02115, USA; 7 Senior author; 8 Lead contact

## Abstract

Epstein-Barr virus (EBV) causes infectious mononucleosis, triggers multiple sclerosis, and is associated with 200,000 cancers/year. EBV colonizes the human B cell compartment and periodically reactivates, inducing expression of 80 viral proteins. However, much remains unknown about how EBV remodels host cells and dismantles key antiviral responses. We therefore created a map of EBV-host and EBV-EBV interactions in B cells undergoing EBV replication, uncovering conserved herpesvirus versus EBV-specific host cell targets. The EBV-encoded G-protein-coupled receptor BILF1 associated with MAVS and the UFM1 E3 ligase UFL1. Although UFMylation of 14-3-3 proteins drives RIG-I/MAVS signaling, BILF1-directed MAVS UFMylation instead triggered MAVS packaging into mitochondrial-derived vesicles and lysosomal proteolysis. In the absence of BILF1, EBV replication activated the NLRP3 inflammasome, which impaired viral replication and triggered pyroptosis. Our results provide a viral protein interaction network resource, reveal a UFM1-dependent pathway for selective degradation of mitochondrial cargo, and highlight BILF1 as a novel therapeutic target.

## In brief

Epstein-Barr virus (EBV) contributes to ~ 2% of human cancers. Yiu et al. assemble an EBV protein interaction network in B cells undergoing viral replication, uncovering that EBV repurposes its G-protein-coupled receptor BILF1 to subvert MAVS-driven NLRP3 inflammasome activation. They highlight UFM1ylation in selective MAVS targeting for lysosomal proteolysis.

## Graphical Abstract

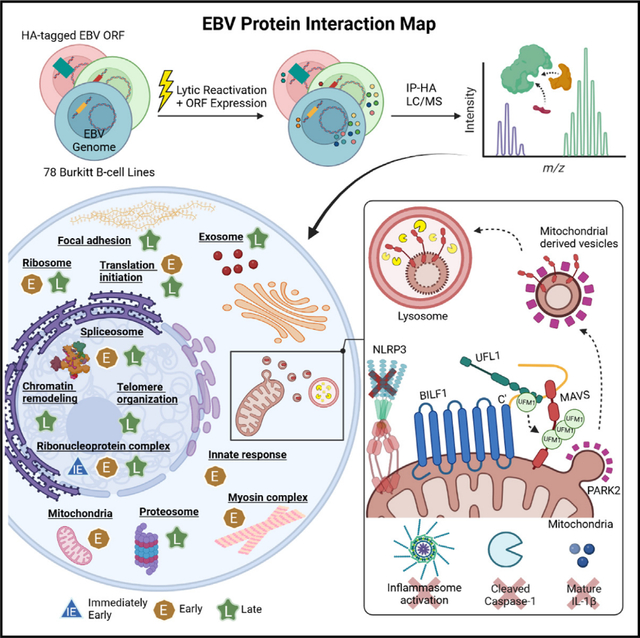

## INTRODUCTION

The γ-herpesvirus Epstein-Barr virus (EBV) establishes lifelong infection in most people worldwide. EBV contributes to ~ 2% of human cancers, including endemic Burkitt lymphoma, Hodgkin lymphoma, and post-transplant lymphoproliferative diseases.^1–5^ EBV also contributes to nasopharyngeal carcinoma and ~ 10% of gastric cancers.^6–8^ EBV is a key trigger for multiple sclerosis.^9^ However, the function of many EBV-encoded proteins remains incompletely understood.

To achieve persistent infection, EBV employs a biphasic life-cycle, in which it alternates between latency and lytic replication. EBV latency programs express between one and nine viral oncogenes that support colonization of the memory B cell compartment. EBV reactivates, although memory B cells differentiate into plasma cells, highlighting the requirement to evade immune detection as EBV spreads to epithelial cells.^10^ Upon reactivation, 80 EBV proteins are expressed in a cascade of immediate early, early, and late genes leading to the synthesis of new viral progeny.^5,11,12^ Immediate early transcription activators BZLF1 and BRLF1 trigger expression of 40 early genes, which replicate EBV DNA and remodel host cells. ~35 late genes contribute to EBV virion assembly and secretion. Despite the label lytic replication, EBV+ B cells undergoing replication remain viable for a period of time as they secrete virion.^5,11–13^

Most EBV infections are asymptomatic, suggesting that EBV has evolved to modulate diverse aspects of host immunity. EBV encodes multiple proteins that mimic immune receptors, cytokines, or chemokines and subverts still other immune pathways to enter B cells.^14,15^ However, much remains to be learned about how EBV dismantles innate and adaptive immunities to hide pathogen-associated molecular patterns (PAMPs) produced during replication.

Although EBV latency proteins drive lymphoproliferative diseases,^16,17^ lytic cycle proteins are increasingly implicated in EBV pathogenesis, including lymphomagenesis.^18,19^ EBV lacking the lytic cycle inducing BZLF1 gene causes lymphomas at reduced frequencies in humanized mouse models.^20^ The lytic cycle BCL-2 homologs BHRF1 and BALF1 suppress apoptosis,^21,22^ whereas BGLF4, BGLF5, and BALF3 destabilize the host genome.^23–25^ The major EBV tegument protein BNRF1 not only destabilizes host SMC5/6 cohesins to support late gene expression^26^ but also causes host chromosome instability.^27^

Here, we assembled an EBV-B cell protein-protein interaction (PPI) network in B cells induced for EBV replication, an environment in which all EBV open reading frames (ORFs) are physiologically expressed. Cross-comparison with human cytomegalovirus (HCMV) and Kaposi sarcoma-associated herpesvirus (KSHV) interactomes^28,29^ revealed common targets. We leveraged the map to identify an unexpected mechanism by which EBV inhibits NLPR3 inflammasome activation. In addition to functioning as a viral G-protein-coupled receptor (GPCR), a subpopulation of EBV BILF1 traffics to mitochondria, where it drives mitochondrial antiviral-signaling protein (MAVS) UFMylation. This signal, together with Parkin ubiquitination, triggers MAVS selective dislocation from the mitochondrial outer membrane, incorporation into mitochondrial-derived vesicles (MDVs), and lysosomal degradation. Our study highlights a novel ubiquitinfold modifier 1 (UFM1)-dependent pathway for mitochondrial membrane cargo turnover, subverted by EBV to enable viral replication and inflammasome evasion.

## RESULTS

### EBV lytic cycle protein interaction map construction

To interrogate EBV PPIs in the lytic B cell environment, we generated 78 P3HR-1 Burkitt lymphoma cell lines, each expressing a doxycycline-inducible EBV ORF. Only EBV LF3 and BHLF1 were not included, due to their extremely high GC content. Lytic reactivation was driven by conditional immediate early BZLF1 and BRLF1 alleles fused to modified estrogen receptor 4-hydroxytamoxifen (4HT)-binding domains (referred to as ZHT and RHT).^30^ 4HT addition triggers ZHT and RHT nuclear translocation and lytic reactivation. We recently used P3HR-1 ZHT/RHT cells for whole-cell EBV lytic cycle proteomic analysis, facilitating cross-comparison.^31^ EBV ORF expression was validated by anti-HA tag immunoblot and flow cytometry (Table S1).

**Figure 1.**
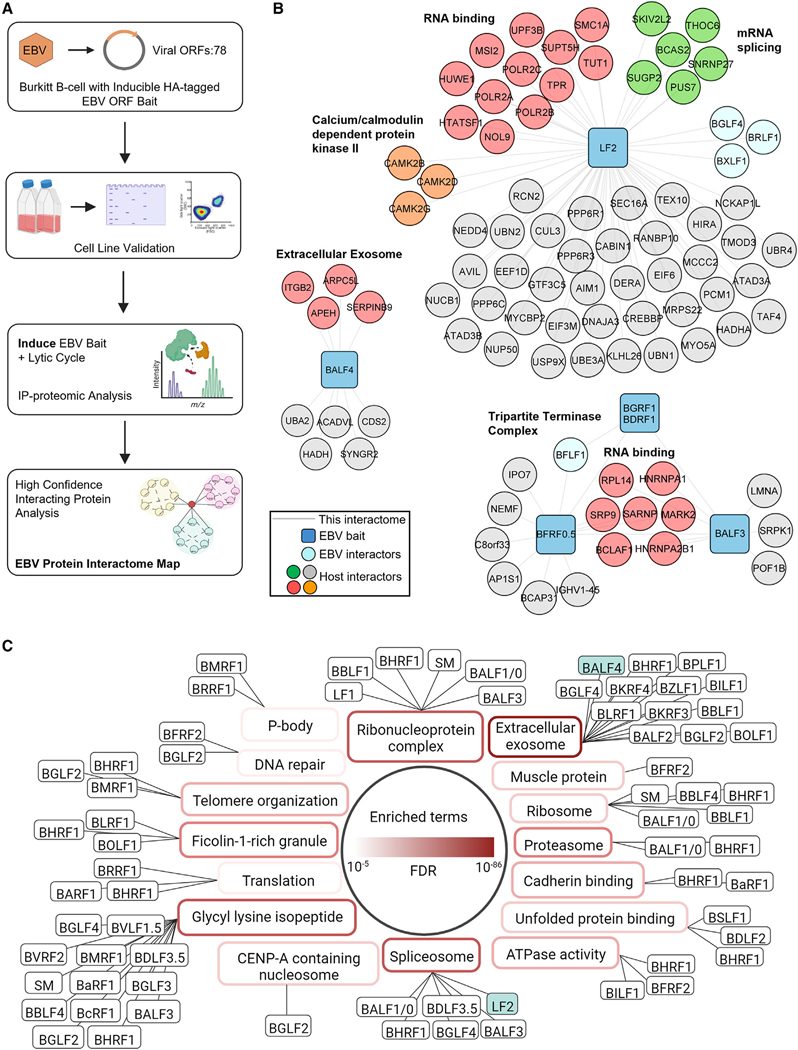
EBV lytic cycle protein interaction map construction (A) EBV protein interaction map workflow. Very high-confidence interacting proteins (VHCIPs) were defined as prey meeting peptide spectral match (PSM) and entropy criteria with either an NWD ≥ 1.0 or Z score ≥ 4.0. High-confidence interacting proteins (HCIPs) were defined as prey meeting PSM and entropy criteria with either an NWD R 1.0 or Z score R 3.0 (Table S2). (B and C) DAVID with default setting was applied to determine pathways enriched among all HCIPs, in comparison to all human proteins as background (full data, Table S3). Benjamini-Hochberg adjusted p values are shown in red with gradient scale to the top 16 unique pathway enriched (p < 0.01). Viral baits were assigned to their top unique enriched pathway with p < 0.05. Viral baits (blue squares), interacting proteins (circles, with viral proteins in light blue), enriched pathway members (red or green), and other host interactors not associated with the pathway (gray) are shown. Black solid lines indicate interactions identified in this study. See also Figure S1.

EBV ORF expression and the viral lytic cycle were induced by the addition of doxycycline and 4HT for just 15 and 24 h, respectively. Anti-HA immunoprecipitation from whole-cell lysates followed by HA peptide elution was performed to isolate EBV protein complexes by liquid chromatography-tandem mass spectrometry (LC-MS/MS) (Figure 1A). High-confidence PPIs were identified by CompPass.^32–34^ Data reported for each prey protein includes: (1) the number of peptide spectral matches (PSMs), averaged between technical replicates; (2) an entropy score, which compares the number of PSM between replicates to eliminate proteins that are not detected consistently; (3) a *Z* score, calculated in comparison to the average and standard deviation of PSMs observed across all IPs; and (4) a normalized WD (NWD) score. The NWD score addresses whether (1) the protein is detected across all IPs and (2) whether it is detected reproducibly among replicates.

Using a stringent cutoff of either an NWD ≥ 1.0 or *Z* score ≥ 4.0, we identified 884 unique viral-host and 83 viral-viral very high-confidence interacting proteins (VHCIPs) (Table S2). Likewise, at a somewhat more relaxed entropy cutoff of either an NWD ≥ 1.0 or *Z* score ≥ 3.0, 1,398 viral-host and 90 viral-viral high-confidence interacting proteins (HCIPs) were identified. EBV baits retrieved 0–285 interactions, yielding a scale-free distribution similar to those reported for two human herpesvirus PPI networks^28,29^ (Figure S1A). Multiple well-characterized EBV-host interactions were identified, providing validation of the proteomic approach and raising confidence in the majority of high-confidence interactions that were not previously reported. For example, consistent with published reports, we identified high-confidence interactions between the viral pre-initiation complex TATT-binding protein BcRF1 and host RNA polymerase II,^35^ between EBV tegument protein BGLF2 and the cell cycle regulatory proteins GMIP and NEK9^36^ (Table S2). Likewise, we captured multiple known high-confidence interactions between EBV proteins, such as between LF2 and the immediate early lytic protein BRLF1,^37–39^ between the viral helicase BBLF4 and primase BSLF1^40^ (Figure 1B; Table S2), and between components of the tripartite terminase and viral pre-initiation complexes (Figure 1B; Table S2). However, most high-confidence EBV-EBV interactions in our dataset are not present in the Biogrid database, highlighting multiple EBV and host protein interaction hubs. For instance, three calcium/calmodulin-dependent protein kinase subunits were identified as VHCIPs of LF2, but not of any other EBV bait (Figure 1B; Table S2).

To gain further insights into host pathways enriched among viral bait interactors, we used database for annotation, visualization, and integrated discovery (DAVID) gene ontology (GO) analysis^41,42^ (Figures 1C and S1B–S1F; Table S3). DAVID indicated that EBV targeting of multiple host pathways not previously implicated in its replication, including the extracellular exosome (Figure 1C). EBV BALF4, which encodes the glycoprotein B (gB) fusogen conserved across herpesvirus cellular entry machinery, associated with exosome cargo (Figures 1B and 1C). Fascinatingly, 12 additional EBV lytic proteins also associated with exosome pathway components, suggesting potentially major exosome pathway roles in EBV egress (Figure 1C; Table S3). Likewise, DAVID highlighted EBV targeting of the mRNA splicing via the spliceosome pathway. Although EBV SM association with ribonucleoprotein complex proteins is well-characterized,^43,44^ five additional EBV lytic protein HCIPs were also enriched for ribonucleoprotein complexes, suggesting that EBV remodels this key pathway to a greater extent than has been appreciated (Figure 1C; Table S3). Glycyl-lysine isopeptide machinery was highly enriched within interactors of 13 EBV proteins, suggesting potentially major EBV roles in subverting ubiquitin, ISG15 and/or SUMO ligases (Figure 1C; Table S3).

### Interactome analysis identifies herpesvirus-targeted host nodes

**Figure 2.**
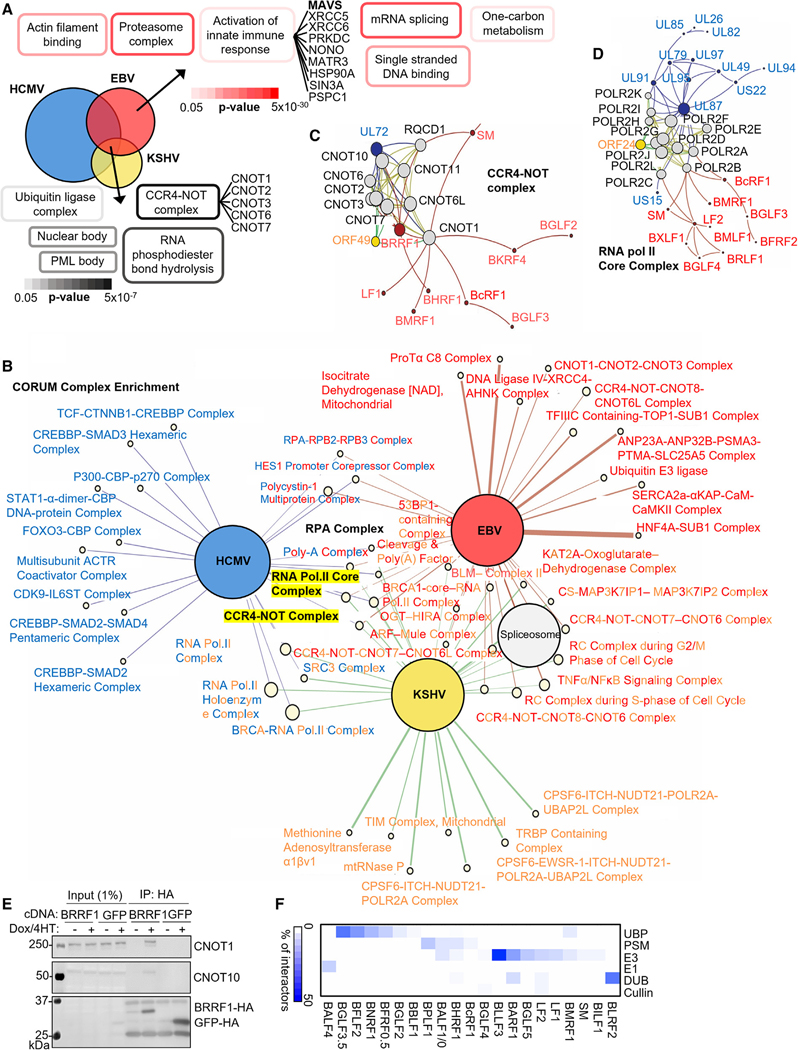
Systematic analysis of interactome data from three herpesviruses highlights common host targets (A) Overlap between EBV, HCMV,^28^ and KSHV^29^ HCIPs (see also Table S4). DAVID pathway enrichment analysis among HCIPs interacting with EBV, HCMV, and KSHV baits (default settings, against all human proteins as background) are shown at bottom, including a list of all commonly interacting CCR4-NOT complex components. Shown at upper right are representative terms from DAVID analysis of HCIPs interacting with EBV proteins but not HCMV or KSHV proteins, and a list of interacting components from the term “activation of innate immune response.” See Table S4 for full Venn diagram details. (B) Network propagation identification of CORUM database human protein complexes that are closely associated with proteins from each herpesvirus. Large colored nodes represent each virus (HCMV, blue; EBV, red; KSHV, yellow). Edges connect to CORUM complexes closely associated with proteins from each virus. Edge thickness is proportional to the Z score observed, whereas CORUM nodes sizes are colored pale yellow and scaled according to the numbers of proteins in each complex. (C and D) Interaction network depicting the CORUM CCR4-NOT and viral protein neighbors. Nodes represent proteins, whereas edges represent protein-protein interactions. Human proteins belonging to the CORUM CCR4-NOT complex (C) or RNA Pol II core complex (D) and edges among them are colored yellow. Viral proteins and their interactions are colored as in (B). Node size is scaled according to each node’s eigenvector centrality within the displayed subnetwork. Graph layouts were determined via gravity embedding. (E) Immunoblot of 1% input and anti-HA complexes from P3HR-1 expressing BRRF1 or GFP cDNAs, representative of n = 3 experiments. (F) Interaction of EBV proteins with components of the cellular degradation machinery. HCIP were searched against a database of E1, E2, E3 enzymes, cullins, deubiquitinases (DUBs), proteasome components (PSM), and ubiquitin-binding proteins (UBPs).^45^ Percentage of the total number of interactors for each protein from each of these categories is displayed. Values were clustered hierarchically (see also Table S5).

All herpesviruses use biphasic lifecycles and share the ability to establish persistent infection. To identify common herpesvirus host protein targets, we leveraged beta-herpesvirus HCMV and gamma-herpesvirus KSHV interactome datasets.^28,29^ Overlap between HCIP highlighted common biological targets of all three herpesviruses, which replicate across a wide range of host types (Figure 2A; Table S4). DAVID analysis of commonly interacting host HCIPs indicated enrichment of the promyelocytic leukaemia (PML) body, nuclear body, ubiquitin ligase complex, RNA phosphodiester bond hydrolysis, and the CCR4/NOT deadenylase complex, a major regulator of mRNA turnover^46^ (Figure 2A).

Next, we merged EBV, HCMV, and KSHV interactomes with the BioPlex 3.0 network of human protein interactions^47^ to derive a multi-species PPI network, viewable at https://wren.hms.harvard. edu/ViroPlex/. We then superimposed complexes from the CORUM database,^48^ a curated repository of experimentally characterized mammalian protein complexes onto the network. We used network propagation to quantify the proximity of each complex to each viral proteome. In addition to the CCR4/NOT deadenylase, CORUM analysis highlighted additional protein complexes targeted by all three human herpesviruses, including the DNA single-stranded binding RPA complex, and the RNA polymerase II complex (Figures 2B–2D). EBV early gene BRRF1 interacted with 7 of the 10 CCR4-NOT components, whereas KSHV ORF49 and HCMV UL72 associate with 6 and 10 CCR4-NOT components, respectively (Figure 2C). We validated the interaction between BRRF1 and CCR4/NOT complex members CNOT1 and CNOT10 by co-immunoprecipitation analysis (Figure 2E).

Ubiquitin ligase complexes were also highly enriched among HCIPs that commonly interacted with EBV-, KSHV-, and HCMV-encoded proteins (Figure 2A). We therefore more fully analyzed EBV PPIs with components of the ubiquitin-proteasome system. HCIPs were searched against a comprehensive database of E1, E2, and E3 enzymes, cullins, deubiquitinases (DUBs), proteasome components (PSM), and ubiquitin-binding proteins (UBPs).^45^ This revealed EBV association with cellular degradation machinery at multiple levels (Figure 2F; Table S5). This identified key “hubs” targeted by multiple herpes viruses.

### BILF1 supports EBV late gene expression and blocks NLRP3 inflammasome activation

**Figure 3.**
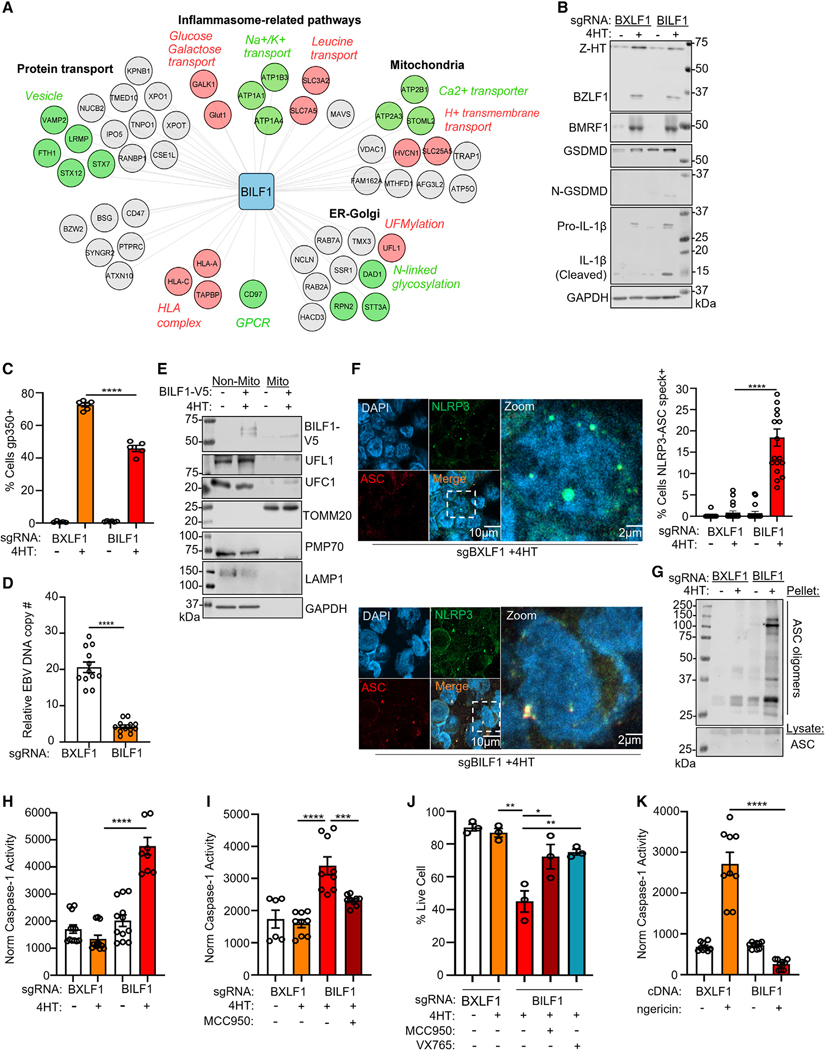
BILF1 inhibits NLRP3 inflammasome activation (A) Cytoscape version 3.8.1 BILF1 interaction network. DAVID pathways enriched among BILF1 interactors are annotated in black. Sub-pathways are annotated either red or green with italicized characters. Host proteins associated with the main or sub-pathways are colored in red or green, remaining proteins are in gray. (B) Immunoblot of WCL from P3HR-1 expressing the indicated sgRNA and uninduced or induced for lytic reactivation by 4HT (400 nM) for 24 h. Representative of n = 3 replicates. (C) Mean ± SEM of plasma membrane (PM) gp350 levels in P3HR-1 expressing the indicated sgRNA, uninduced or 4HT-induced for reactivation for 24 h. (D) Quantitative real-time PCR (qRT-PCR) of EBV intracellular genome copy number from P3HR-1 with the indicated sgRNA and 4HT-induced for 24 h. Mean ± SEM from n = 3 replicates. (E) Immunoblot of 2.5% input and anti-HA immunopurified mitochondria from P3HR-1 stably expressing HA-OMP25, uninduced or 4HT-induced for 24 h. Representative of n = 2 replicates. (F) Immunofluorescence analysis of NLRP3 and ASC in P3HR-1 expressing the indicated sgRNA, 4HT-induced for 24 h. Right: mean ± SEM percentage of cells with NLRP3/ASC specks from n = 3 replicates, as in (E), using data from 20 randomly selected panels of 200 nuclei, analyzed by ImageJ ComDet plugin. (G) Immunoblot of ASC oligomerization from P3HR-1 expressing the indicated sgRNA, uninduced or 4HT-induced for 24 h. Representative of n = 2 replicates. (H) Mean ± SEM from n = 3 replicates of caspase-1 activity normalized by live cell number from P3HR-1 expressing the indicated sgRNA and 4HT-induced for 24 h. (I) Mean ± SEM from n = 3 replicates of caspase-1 activity normalized by live cell number from P3HR-1 cells expressing the indicated sgRNA, 4HT-induced ± the NLRP3 inhibitor MCC950 (10 μM) for 24 h. (J) Mean ± SEM live cell percentages from n = 3 replicates of trypan blue staining of P3HR-1 expressing the indicated sgRNA and treated with 4HT, MCC950 (10 μM) or the caspase/pyroptosis inhibitor VX795 (10 μM) for 24 h. (K) Mean ± SEM from n = 3 replicates of caspase-1 activity normalized by live cell number from P3HR-1 expressing the indicated cDNA and nigericin treated for 24 h. Student’s t test was performed, with ****p < 0.0001, ***p < 0.001, **p < 0.01, *p < 0.05. See also Figure S2.

Much remains to be learned about how EBV evades immune responses to periodically reactivate. We were therefore intrigued that the EBV-encoded 7-transmembrane protein orphan GPCR BILF1 interacted with host factors involved in innate and adaptive immune responses, metabolism, and transport (Figure 3A). BILF1 is highly conserved across lymphocryptoviruses and is expressed early in the lytic cycle.^49^ Consistent with its roles in evasion of the human leukocyte antigen (HLA) presentation pathway,^50,51^ we identified high-confidence interactions between BILF1 and multiple HLA alleles. We also identified an association between BILF1 and the TAP-binding protein (TAPBP), which mediates an association between the TAP peptide transporter and class I molecules awaiting peptide cargo (Figure 3A). BILF1 may therefore subvert antigen presentation within the endoplasmic reticulum as well as at the plasma membrane (PM), perhaps together with BNLF2a, which blocks TAP.^52^

Given the diversity of BILF1 host and viral protein interactions identified, we tested BILF1 knockout (KO) effects on EBV replication. Since an antibody against *BILF1* is not available, high-frequency *BILF1* CRISPR editing in latent P3HR-1 cells was validated by insertion or deletion (indel) sequencing and by immunoblot of exogenous V5-tagged BILF1 depletion (Figures S2A and S2B). A single-guide RNA (sgRNA) against EBV lytic gene *BXLF1,* which is not essential for replication,^26,53^ was performed as a control for EBV genome editing. BILF1 KO had little effect on EBV immediate early BZLF1 or early BMRF1 expression, but impaired late gene gp350 expression (Figures 3B, 3C, and S2C). BILF1 KO also reduced lytic EBV genome copy number and infectious virion production by approximately 75% and 50%, respectively (Figures 3D and S2D). Given BILF1 roles in support of both immune evasion and viral replication, we decided to pursue BILF1 function in depth.

Our proteomic analysis identified that BILF1 and BHRF1, but not other EBV proteins interacted with MAVS, a key pattern recognition receptor (PRR) (Figure 3A; Table S2). MAVS activates downstream interferon and inflammasome pathways in response to retinoic acid-inducible-I (RIG-I) and melanoma differentiation-associated gene 5 (MDA5), typically in response to viral RNA PAMPs. Little has remained known about how EBV evades inflammasome pathways, which can exert potent antiviral effects. Therefore, given the proteomic signals that BILF1 associates with MAVS and other proteins potentially related to the inflammasome pathway activators (Figure 3A), we investigated potential BILF1 MAVS evasion roles. We first validated that a subpopulation of BILF1 interacts with MAVS and localizes to mitochondria. HA-tagged BILF1 co-immunoprecipitated MAVS from lysates of cells undergoing EBV lytic replication (Figure S2E). Similarly, we identified that a BILF1 subpopulation co-localized with the mitochondrial outer membrane protein TOMM20 (Figure S2F) and with immunopurified mitochondria (Figure 3E).

A key scaffold MAVS role in support of NLRP3 activation was reported.^54^ However, little has remained known about how EBV evades antiviral inflammasome pathways. As the EBV kinase BGLF4 suppresses interferon regulatory factor 3 (IRF3) responses downstream of MAVS,^55^ we hypothesized that BILF1 blocks MAVS to prevent NLRP3 inflammasome activation. To investigate this, we analyzed NLRP3 inflammasome assembly in control versus BILF1 KO B cells induced for reactivation. Although NLRP3 levels mildly increased upon EBV reactivation (Figure S2G), discrete foci of NLRP3 and the apoptosis-associated speck-like protein 2 (ASC2) co-localized, indicative of NLRP3 inflammasome activation (Figure 3F). We also observed the formation of high molecular weight ASC2 oligomers, indicative of NLRP3 inflammasome assembly (Figure 3G). Since the PRR absent in melanoma-2 (AIM2) assembles inflammasomes in response to murine cytomegalovirus DNA,^56^ we next tested EBV effects on AIM2. In contrast to effects on NLRP3, EBV reactivation did not induce co-localization between AIM2 and ASC, even with BILF1 KO (Figures S2H and S2I).

NLRP3 inflammasomes process IL-1β, IL-18, and gasdermin D (GSDMD) to induce pro-inflammatory signaling and pyroptosis cell death.^57–59^ We asked whether BILF1 restrains caspase-1 activation downstream of the NLRP3 inflammasome. Immunoblot analysis detected GSDMD and IL-1β caspase cleavage products in BILF1 KO, but not BXLF1 KO control P3HR-1 undergoing replication (Figure 3B). Similarly, caspase-1 activity was significantly increased in lytic BILF1 KO cells (Figure 3H), which was reduced by the NLRP3 inhibitor MCC950^60^ (Figure 3I). Similarly, treatment with either MCC950^61^ or the caspase-1/4 inhibitor VX765^62,63^ significantly increased live cell number in lytic BILF1 KO cells (Figure 3J).

Given that BILF1 associated with Na+/K+ transporting ATPase catalytic ATP1A1 and non-catalytic ATP1B3 subunits (Figure 3A), we tested whether BILF1 could block NLRP3 inflammasome assembly induced by the potassium ionophore nigericin. Constitutive expression of BILF1, but not BXLF1 cDNA, impaired NLRP3/ASC speck assembly, caspase-1 activity, and production of cleaved GSDMD and IL-1β in Akata cells (Figures 3K and S2J–S2L). Collectively, these results suggest that BILF1 subverts NLRP3 inflammasome activation otherwise triggered by EBV lytic replication.

### BILF1 dislocates MAVS from the mitochondria to inhibit inflammasome activation

**Figure 4.**
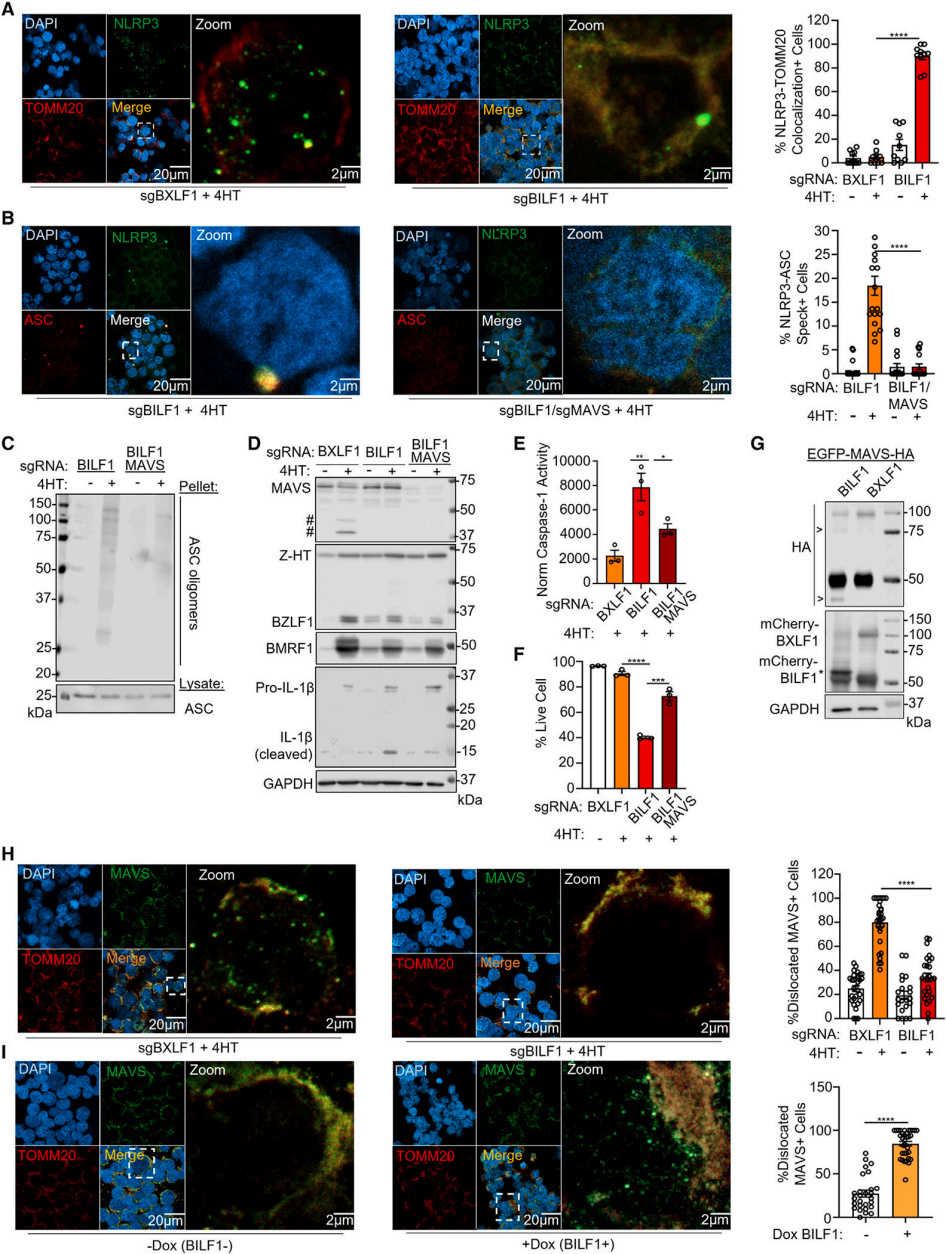
BILF1 mediates MAVS dislocation from the mitochondria to inhibit NLRP3 inflammasome activation (A) Immunofluorescence analysis of NLRP3 and TOMM20 in P3HR-1 expressing the indicated sgRNA, 4HT-induced for 24 h. Right: mean ± SEM percentage of cells with NLRP3-TOMM20 co-localization from n = 3 replicates, using data from 10 randomly selected panels of 200 nuclei, analyzed by ImageJ ComDet plugin. (B) Immunofluorescence analysis of NLRP3 and ASC speck formation in P3HR-1 expressing the indicated sgRNA and 4HT-induced for 24 h. Right: mean ± SEM percentage of cells with NLRP3/ASC specks from n = 3 replicates, using data from 20 randomly selected panels of 200 nuclei, analyzed by ImageJ ComDet plugin. (C) Immunoblot of ASC oligomerization from P3HR-1 expressing the indicated sgRNA, uninduced, or 4HT-induced for 24 h. Representative of n = 2 replicates. (D) Immunoblot of WCL from P3HR-1 expressing the indicated sgRNA, uninduced, or 4HT-induced for 24 h. # indicates low molecular weight bands immunoreactive with anti-MAVS antibody. (E) Mean ± SEM from n = 3 replicates of caspase-1 activity normalized by live cell number from P3HR-1 expressing the indicated sgRNA, 4HT-induced for 24 h. (F) Mean ± SEM from n = 3 replicates of trypan blue analysis of P3HR-1 expressing the indicated sgRNA, 4HT-induced for 24 h. (G) Immunoblot of 293T transiently expressing the indicated cDNA. Representative of n = 2. (H) Immunofluorescence analysis of MAVS and TOMM20 in P3HR-1 expressing the indicated sgRNA, 4HT-induced for 24 h. Right: mean ±SEM percentage of cells with delocalized MAVS from n = 3 replicates, using data from 20 randomly selected panels of 400 nuclei, analyzed by ImageJ ComDet plugin. (I) Immunofluorescence analysis of MAVS and TOMM20 in P3HR-1, ± BILF1 cDNA induced by 5 mM doxycycline for 24 h. Right: mean ± SEM percentage of cells with delocalized MAVS from n = 3 replicates, using data from 30 randomly selected panels of 600 nuclei, analyzed by ImageJ ComDet plugin. Student’s t test was performed, with ****p < 0.0001. ***p < 0.001. **p < 0.01. *p < 0.05. See also Figure S3.

Subcellular mitochondrial or peroxisomal MAVS localization is crucial for NLRP3 inflammasome oligomerization and activation.^54,64–66^ In latent P3HR-1 and Akata B cells expressing BXLF1 or BILF1 sgRNAs, MAVS signal highly overlapped with the mitochondrial outer membrane translocase TOMM20 (Figure S3A). We hypothesized that BILF1 blocks inflammasome activation by interfering with NLPR3 recruitment to mitochondrial membrane MAVS foci. In support, minimal NLRP3 and TOMM20 co-localization was observed in 4HT-induced P3HR-1 expressing control BXLF1 sgRNA. However, BILF1 sgRNA expression resulted in a high degree of NLRP3 and TOMM20 signal overlap upon reactivation, suggesting that BILF1 interferes with NLRP3 mitochondrial recruitment, not previously reported in B lymphocytes (Figure 4A). Furthermore, CRISPR KO of both MAVS and BILF1 prevented NLRP3-ASC speck assembly and ASC oligomerization upon P3HR-1 lytic induction (Figures 4B and 4C). EBV lytic cycle-driven caspase-1 activity, IL-1β processing, and cell death were diminished in BILF1 KO cells by combined MAVS KO (Figures 4D–4F). Thus, despite emerging evidence that NLRP3 inflammasomes are assembled at the centrosome in macrophages,^67,68^ our studies highlight key mitochondrial membrane MAVS roles in NLRP3 inflammasome assembly at mitochondrial membranes in response to B cell EBV lytic induction but raise the question of how a viral 7TM GPCR can block MAVS/NLRP3 signaling.

We next characterized MAVS fate upon EBV lytic induction in control vs. BILF1 KO B cells. Interestingly, several low molecular weight polypeptides reactive with anti-MAVS antibody were evident upon lytic induction of P3HR-1 expressing BXLF1 control, but not BILF1 sgRNA (Figure 4D). MAVS sgRNA depleted these bands, even in cells co-expressing BILF1 sgRNA. The smaller MAVS species were recognized by a polyclonal antibody raised against full-length MAVS, but not by a monoclonal antibody against MAVS residues 34–96 (Figure S3B). Thus, BILF1 induces cleavage of the MAVS N terminus. To investigate this further, we expressed N-terminally EGFP-tagged and C-terminally HA-tagged MAVS (N′-EGFP-MAVS-C′-HA) with monomeric Cherry (mCherry)-tagged BILF1 or control BXLF1 in 293T cells. We note that mCherry-BILF1 migrates at a lower than predicted molecular weight, perhaps because of effects on BILF1 glycosylation. Expression of BILF1, but not BXLF1, resulted in appearance of MAVS fragments reactive with anti-HA antibody at ~ 70 kDa and just below 50 kDa (Figure 4G).

We next performed immunofluorescence microscopy on control versus BILF1 KO cells induced for lytic replication. In P3HR-1 expressing control BXLF1 sgRNA, 4HT treatment caused MAVS redistribution, with the formation of multiple puncta with limited TOMM20 overlap. However, MAVS remained highly TOMM20 co-localized in BILF1 KO P3HR-1 (Figure 4H). Similar results were obtained in Akata cells triggered for reactivation by immunoglobulin cross-linking (Figure S3C). MAVS puncta formation was also evident in lytic EBV+ gastric carcinoma AGS cells (Figure S3D). Therefore, immunoblot persistence of a population of full-length MAVS is likely due to a combination of MDV sequestration and MAVS re-synthesis. Alternatively, a subpopulation may be targeted, for example, multimerized MAVS.

BILF1 expression was sufficient to trigger MAVS dislocation (Figure 4I). We hypothesized that BILF1 might therefore perturb RIG-I-MAVS-driven IRF3 responses. Indeed, EBV lytic cycle-driven caspase-1 activity and cell death in BILF1 KO cells were significantly diminished by combined RIG-I KO (Figures S3E–S3G). IRF3 nuclear translocation and IRF3 puncta formation were observed in BILF1 KO, but not control cells (Figure S3H). EBV BHRF1 was reported to induce mitochondrial fission, induce mitophagy, and inhibit IRF3 nuclear translocation.^69^ Although our proteomic analysis identified BHRF1/MAVS association, neither BHRF1 KO nor BHRF1 cDNA expression altered MAVS localization (Figures S4A and S4B).

### The BILF1 C-terminal tail recruits E3 ligase UFL1 to trigger MAVS K461 UFMylation

**Figure 5.**
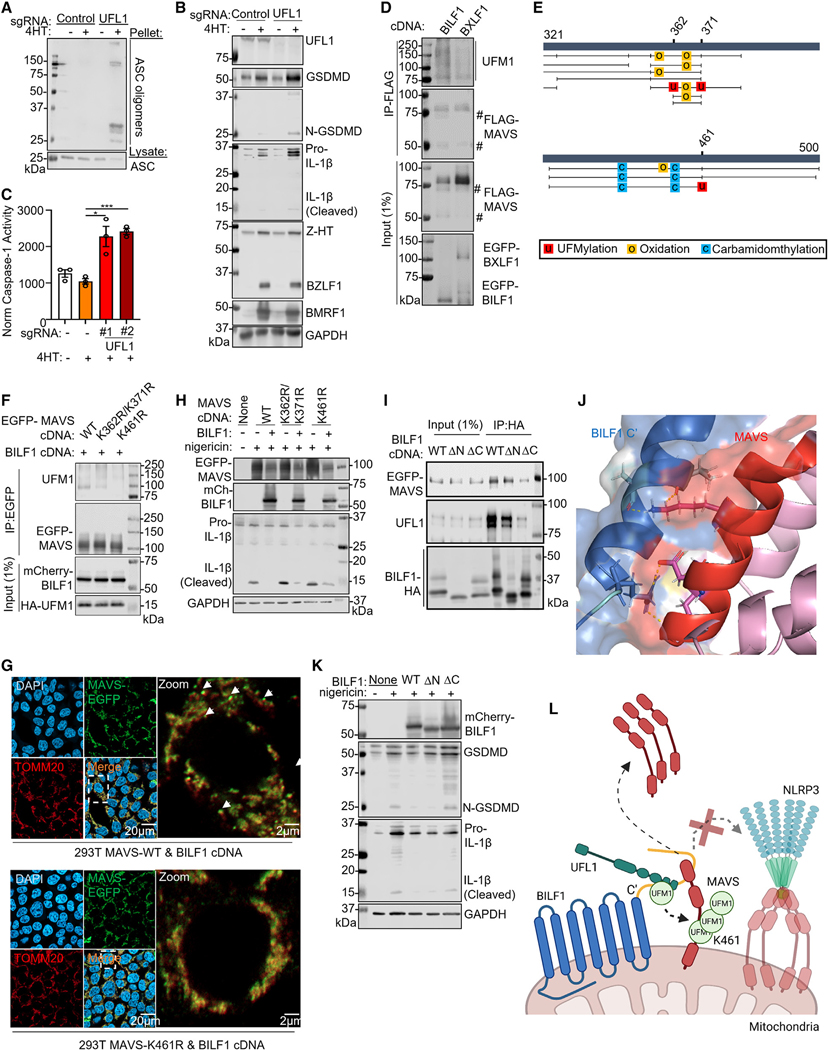
BILF1 triggers MAVS mitochondrial dislocation through UFMylation (A) Immunoblot of ASC oligomerization from P3HR-1 expressing the indicated sgRNA, uninduced or 4HT-induced for 24 h. (B) Immunoblot analysis of WCL from P3HR-1 expressing the indicated sgRNA and 4HT-induced, as indicated. (C) Mean ± SEM caspase-1 activity normalized by live cell number from n = 3 replicates of P3HR-1 expressing the indicated sgRNA and 4HT-treated for 24 h, as indicated. (D) Immunoblot of 1% input and anti-FLAG-MAVS complexes from 293T co-transfected with FLAG-MAVS, BILF1, or BXLF1 cDNAs for 24 h, as indicated. (E) PEAKS software identification of potential MAVS post-translational modification sites. Putative UFMylation sites were identified at lysines 362, 371, and 461. MAVS residues 321–500 are depicted. Black vertical lines represent individual peptide sequencing events. (F) Immunoblot of anti-EGFP-MAVS immunopurified from 293T co-transfected with the indicated MAVS, BILF1, and UFM1 cDNAs for 24 h. (G) Immunofluorescence analysis of wild type or K461R MAVS subcellular localization in 293T co-transfected with MAVS and BILF1 cDNAs for 24 h. (H) Immunoblot of WCL from 293T expressing MAVS and BILF1 cDNA, nigericin stimulated for 24 h, as indicated. (I) Immunoblot of 1% input and anti-HA immunopurified EGFP-MAVS and UFL1 from 293T transfected with BILF1 and EGFP-MAVS cDNAs for 24 h, as indicated. (J) AlphaFold multimer model highlighting the predicted BILF1 and MAVS interaction domain and residues. (K) Immunoblot of WCL from 293T expressing wild type, N- (ΔN) or C- (ΔC) terminal tail deletion mutant BILF1, nigericin stimulated for 24 h, as indicated. (L) Schematic of BILF1 NLRP3 inflammasome inhibition. Student’s t test was performed, with ***p < 0.001. *p < 0.05. See also Figures S4 and S5. Immunoblots are representative of n = 2.

Proteomic analysis identified high-confidence interaction between BILF1 and host UFM1 specific ligase 1 (UFL1) (Figure 3A), an E3 ligase that mediates covalent attachment of the ubiquitinlike modifier UFM1 to target protein lysine residues in a process termed UFMylation.^70^ We validated this result by co-immunoprecipitation analysis (Figure S2E). Likewise, immunoprecipitation analysis identified that small populations of UFL1 and UFMylation pathway E2 ligase UFC1 were recruited to mitochondria in cells undergoing lytic replication (Figure 3E). Time-course analysis highlighted the appearance of a high molecular weight MAVS species migrating just above the full-length MAVS band, whose abundance was diminished by BILF1 KO (Figure S4C). We hypothesized that the high-molecular weight species was UFMylated MAVS and established control versus UFL1 KO P3HR-1. Although NLRP3 and ASC did not co-localize in 4HT-treated controls, NLRP3/ASC specks and ASC polymerization were evident in 4HT-treated UFL1 KO P3HR-1 (Figures 5A, S4D, and S4E). UFL1 KO also increased caspase-1 activity, GSDMD, and IL-1b processing in reactivated cells (Figures 5B and 5C). Likewise, UFL1 KO diminished the appearance of MAVS cleavage products (Figure S4F).

We next investigated whether BILF1 expression was sufficient to trigger MAVS UFMylation. MAVS co-immunoprecipitated with UFL1 in cells expressing BILF1, but not BXLF1 (Figure S4G). Furthermore, BILF1 co-expression with C-terminal FLAG-tagged MAVS triggered the appearance of low molecular weight bands reactive with anti-FLAG antibody, suggestive of N-terminal MAVS cleavage. BILF1 expression also induced the formation of high molecular weight bands recognized by anti-UFM1 antibody on immunoblot analysis of purified FLAG-MAVS complexes (Figure 5D). Since samples were boiled prior to immunoprecipitation to disrupt protein complexes, this result is suggestive of covalent UFMylation.

To identify specific UFMylation site(s), we analyzed MAVS complexes immunopurified from BILF1+ vs. BXLF1+ cells by LC/MS. UFMylation signals were identified by the PEAKS algorithm at MAVS lysines K362/K371 and K461 only in BILF1+ cells.^71^ The absence of trypsin cleavage sites between K362 and K371 prevented assignment to just one of these lysines. We also used the CORE algorithm, which identified UFMylation signals only in BILF1+ cells at MAVS K461 (Figure 5E). To extend these MS results, we expressed MAVS lysine to arginine K362R/K371R and K461R point mutants together with BILF1. High-molecular-weight UFM1 signal of immunopurified K461R, but not K362R/K371R MAVS, suggested that BILF1 drives MAVS lysine 461 UFMylation (Figure 5F).

To test if UFMylation was necessary for MAVS mitochondrial dislocation, we knocked out the UFM1 E2 and E3 ligases, UFC1 and UFL1. Depletion of either impaired EBV MAVS dislocation (Figures S4H and S4I). Also suggestive of a key UFMylation role, BILF1 triggered dislocation of wild type and K362R/K371R MAVS, but not K461R in 293T (Figures 5G and S5A), and BILF1 blockade of nigericin NLRP3 inflammasome activation required UFL1 (Figures S5B and S5C). However, K461R MAVS expression precluded BILF1 NLRP3 antagonism (Figures 5H and S5D). We next asked if BILF1-mediated UFMylation impacted MAVS activation of downstream interferon responses. We overexpressed MAVS in 293T, which causes its oligomerization and activates downstream signaling.^72^ However, BILF1 blocked MAVS induction of the interferon-stimulated gene IFIT1 in a UFL1-dependent manner (Figure S5E).

Finally, we sought to identify the BILF1 region that associates with MAVS and UFL1. AlphaFold modeling^73,74^ suggested potential interactions between the BILF1 C-terminal tails, MAVS and UFL1 (Figures 5J and S5F). We generated BILF1 N- (ΔN) or C- (ΔC) terminal tail deletion mutants and performed co-immunoprecipitation analysis. ΔC-BILF1 had a reduced association with MAVS and UFL1 (Figure 5I) and diminished MAVS mitochondrial dislocation (Figure S5G). We note that BILF1 over-expression in 293T resulted in a low level of glycosylation, such that most BILF1 migrated at the predicted non-glycosylated molecular weight. DC-BILF1 also failed to inhibit nigericin NLRP3 activation (Figures 5K and S5H). By contrast, BILF1 C174A and K122A point mutants,^75^ which abolish GPCR signaling and MHC class I downregulation, did not affect MAVS dislocation or NLRP3 inflammasome inhibition (Figures S5I and S5J). These data suggest key BILF1 C-terminal tail roles in MAVS K461 UFMylation (Figure 5L).

### BILF1 dislocates MAVS into MDVs for lysosomal proteolysis

**Figure 6.**
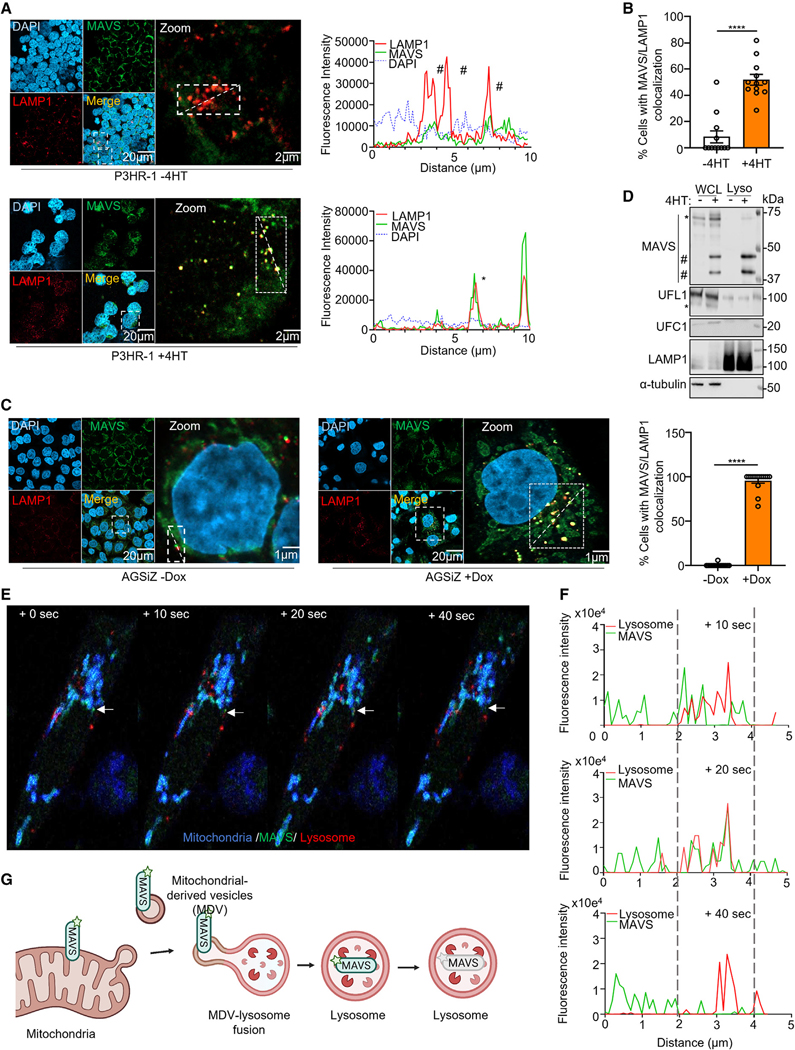
MAVS sequestration and lysosomal fusion events captured in real time (A) Left: immunofluorescence analysis of MAVS and LAMP1 in P3HR-1, uninduced, or 4HT-induced for 24 h. Right: fluorescence intensity line scanning of LAMP1 (red) and MAVS (green) in the white rectangle. * and # mark co-localization and non-co-localization, respectively. (B) Mean ± SEM percentage of cells with MAVS-LAMP1 co-localization from n = 3 replicates, as in (A), using data from 12 randomly selected panels of 240 nuclei, analyzed by ImageJ ComDet plugin. (C) Immunofluorescence analysis of MAVS and LAMP1 in EBV+ AGSiZ gastric carcinoma, lytic induced by doxycycline for 24 h. Right: mean ± SEM percentage of cells with MAVS-LAMP1 co-localization from n = 3 replicates, using data from 12 randomly selected panels of 240 nuclei, analyzed by ImageJ ComDet plugin. (D) Immunoblot of 2.5% input and anti-HA immunopurified lysosomes from P3HR-1 HA-TMEM192+ cells, 4HT-induced for 24 h, as indicated. High and low molecular weight bands reactive with anti-MAVS antibodies are denoted by #. UFL1 is denoted by *. Representative of n = 2. (E) Consecutive frames captured at 10 s intervals at 3.5 h post AGSiZ lytic induction by doxycycline. White arrows highlight a GFP-MAVS puncta (green) superimposed on BFP-labeled mitochondria (blue) and SIRylo-stained lysosomes (red). Consecutive frames show partial co-localization of the GFP-MAVS puncta and lysosome (red) signals at +10 and +20 s, and then loss of MAVS puncta signal at the site of lysosome overlap at +40 s. See also corresponding Video S1. (F) Fluorescence intensity line scanning of lysosome SIRylo (red) and GFP-MAVS (green) signals at the white arrow marked puncta in (F) at +10, +20, and +40 s. (G) Schematic illustration of UFMylated MAVS trafficking from mitochondria to lysosome via MDVs. Student’s t test was performed, with ****p < 0.0001. See also Figure S6 and Video S1.

To gain insights into MAVS disposition in BILF1+ cells, we performed confocal microscopy. 4HT triggered MAVS co-localization with the lysosomal marker LAMP1 in P3HR1 and AGS cells (Figures 6A–6C, S6A, and S6B). We therefore asked whether MAVS N-terminal cleavage fragments were enriched within immunopurified lysosomes.^76^ Immunoblots of cytosolic tubulin versus lysosomal LAMP1 controls indicated a high degree of lysosomal enrichment (Figure 6D). Importantly, the lysosomal fraction contained predominantly MAVS N-terminal cleavage products, whereas full-length and high-molecular-weight MAVS were instead more abundant in whole-cell lysate (WCL). We did not observe an enrichment of either UFC1 or UFL1 in lysosome fractions, suggesting that MAVS is delivered to lysosomes in the absence of other UFMylation pathway components (Figure 6E).

MDVs transport cargo generated by selective incorporation of mitochondrial content.^77–81^ Although these can include mitochondrial outer membrane proteins, MAVS incorporation into MDVs has not been described. Therefore, to investigate MAVS trafficking, we stably expressed N-terminally GFP-tagged MAVS and then transiently expressed mitochondrial-targeted *Aequorea victoria* blue fluorescence protein (BFP) in AGSiz cells. Lysosomes were imaged by live cell staining with the SiR lysosomal dye, which is activated by lysosomal cathepsins. Beginning at approximately 200 min post-induction, we observed GFP-MAVS signal budding off mitochondrial membranes (Figure 6E). MAVS-GFP signal then co-localized with SiR, with rapid subsequent loss of GFP signal, consistent with lysosomal destruction (Figures 6E and 6F; Video S1). These data support a model in which MAVS traffics via MDVs to lysosomes (Figure 6G).

### BILF1 utilizes PARK2 for lysosomal MAVS dislocation

**Figure 7.**
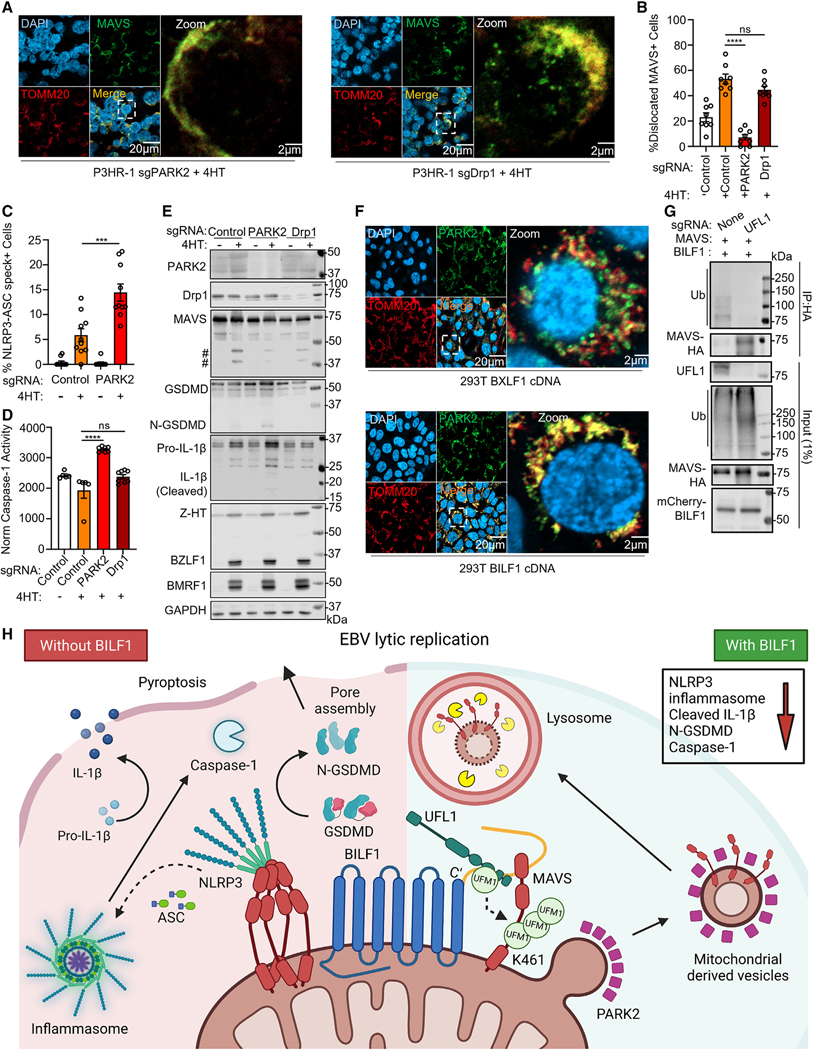
BILF1 dislocates MAVS to mitochondria derived vesicles. (A) Immunofluorescence analysis of MAVS and TOMM20 in P3HR-1 expressing the indicated PARK2 or Drp1 sgRNAs and 4HT-induced for 24 h. (B) Mean ± SEM percentage of cells with dislocated MAVS from n = 3 replicates, as judged by appearance of MAVS puncta that did not overlap with TOMM20 signal as in (A), using data from 25 randomly selected panels of 500 nuclei, analyzed using ImageJ ComDet plugin. (C) Mean ± SEM percentage of P3HR-1 with NLRP3/ASC specks from n = 3 replicates, using data from 20 randomly selected panels of 200 nuclei, analyzed by ImageJ ComDet plugin. (D) Mean ± SEM caspase-1 activity normalized by live cell number from n = 3 replicates of P3HR-1 expressing the indicated sgRNA and 4HT-induced for 24 h, as indicated. (E) Immunoblot of WCL from P3HR-1 expressing the indicated sgRNA and 4HT-induced for 24 h. # denotes low molecular weight bands immunoreactive with anti-MAVS antibody. Representative of n = 3. (F) PAKR2 and TOMM20 immunofluorescence analysis in 293T transfected with BILF1 or BXLF1 cDNA for 24 h. (G) Immunoblot of 1% input vs. anti-HA-MAVS immunopurified from wild type or UFL1-KO 293T transfected with MAVS and BILF1 cDNA for 24 h, as indicated. Representative of n = 2. (H) Schematic of NLRP3 inflammasome subversion by BILF1. BILF1 recruits UFL1 to mediate MAVS UFMylation, which together with PARK2 triggers selective MAVS removal from the mitochondrial outer membrane, MDV packaging and delivery to lysosomes, preventing NLRP3 inflammasome activation and pyroptosis. Student’s t test was performed, with ****p < 0.0001. ***p < 0.001. ns > 0.05. See also Figure S6.

UFMylation is implicated in selective lysosomal autophagic turnover of endoplasmic reticulum contents.^82^ We hypothesized that BILF1/UFL1-driven UFMylation could serve to recruit PARK2 (also called PARKIN), an E3 ubiquitin ligase with MDV generation roles in response to mitochondrial stress.^83,84^ We established control and PARK2 KO P3HR-1 and as a further control, P3HR-1 knocked out for the mitophagy regulator Drp1. Dislocated MAVS puncta were evident in reactivated Drp1 KO cells but not PARK2 KO (Figures 7A and 7B). PARK2 KO also significantly increased levels of NLRP3/ASC specks, ASC oligomerization, caspase-1 activity, GSDMD and IL-1b cleavage (Figures 7C, 7D, and S6C), and MAVS fragments (Figure 7E), suggesting a key PARK2 role in MAVS lysosomal delivery.

We hypothesized that PARK2 is recruited to mitochondria to ubiquitylate MAVS, perhaps following UFMylation. To investigate this, we stained PARK2 and TOMM20 in BILF1+ or BXLF1+ 293T. PARK2 co-localized with TOMM20 only with BILF1 expression (Figure 7F). To test if UFMylation was required, we co-expressed BILF1 and MAVS in control vs. UFL1 KO 293T. Immunoblot revealed high-molecular-weight ubiquitin conjugates of MAVS complexes immunopurified from control, but not UFL1 KO (Figure 7G).

Our proteomic analysis highlighted BILF1 association with the Ras-related proteins Rab2A and Rab7A (Figure 3A), which regulate intracellular vesicles transport. Rab2A localizes to autophagosomes,^85^ whereas Rab7A controls late endosome-lysosome fusion.^86^ Confocal analysis highlighted partial co-localization of dislocated MAVS puncta with Rab5A and Rab7A, but not Rab27B or the autophagy marker LC3B in 4HT-treated P3HR-1 (Figures S6D–S6G). This pattern suggests MAVS sorting to endosomes, independently of exosome or autophagy pathways. Collectively, our results support a model in which BILF1 triggers MAVS UFMylation, PARK2-dependent sorting to MDVs, and then lysosome degradation, highlighting a pathway for selective dislocation of mitochondrial membrane cargo (Figure 7H).

## DISCUSSION

We leveraged the first lytic cycle EBV protein interaction map to identify high-confidence B cell and viral protein interactors of nearly all EBV ORFs, many of which have remained little studied. We also presented the Viroplex resource, in which EBV, KSHV, and HCMV interaction networks are presented for cross-comparison. This approach led to the observation that EBV BILF1 hijacks the UFMylation pathway to target MAVS for MDV trafficking and lysosomal degradation, highlighting a means to selectively degrade mitochondrial membrane cargo and prevent NLRP3 inflammasome activation.

In addition to characterized roles in the blockade of CXCR4^87^ and MHC class I,^50,51^ we found that BILF1 targets MAVS to block NLRP3 inflammasomes and IRF3 nuclear translocation. These results raise the question of what MAVS and NLRP3 each sense to culminate in MAVS/NLRP3 association at mitochondrial membrane sites, necessitating BILF1 evasion. HSV and EBV replication unmask a host cell 5S rRNA pseudogene that binds and activates RIG-I.^88^ However, this 5S rRNA sensing pathway activated pro-inflammatory cytokine expression even in BILF1+ cells, suggesting an alternative stimulus may activate MAVS without BILF1.

EBV lytic replication produces multiple RNA species, including circular RNAs,^89,90^ which can activate RIG-I/MAVS.^91^ NLRP3 responds to stress induced by danger-associated molecular patterns,^92^ including alterations in levels of ATP, potassium, sodium, oxidized mitochondrial DNA, or mitochondrial reactive oxygen species. It is noteworthy that our proteomic analysis identified high-confidence interactions between BILF1 and MAVS and between BILF1 and PM transporters of sodium, potassium, glucose, and leucine, raising the possibility that BILF1 may perturb upstream aspects of NLRP3 activation. These proteomic signals were a major impetus to study BILF1 roles in inflammasome evasion.

In response to RNA viral infection, MAVS serves as a key mitochondrial docking site and driver of NLRP3 oligomerization.^54,64,65^ Whether this phenomenon also occurs in response to DNA virus infection has remained unknown. Our findings indicate that MAVS likewise plays key roles in NLRP3 recruitment to mitochondrial outer membranes and in NLRP3 inflammasome activation in response to EBV and therefore likely also to other DNA viruses. Although plasma cell differentiation is the major EBV reactivation trigger, high extracellular glucose can also trigger NLRP3 inflammasomes and EBV lytic reactivation.^93^ Once induced, BILF1 then blocks NLRP3, presumably to prevent pyroptosis, support viral replication, and dampen pro-inflammatory signaling.

Several herpesviruses activate NLRP inflammasomes, highlighting a key difference with EBV. KSHV ORF45 induces conformational changes that drive NLRP1 inflammasome assembly.^94^ This may be balanced by the KSHV ORF63 NLRP1 homolog, which inhibits NLRP1.^95^ However, we speculate that KSHV- and HCMV-encoded GPCRs may not target MAVS, given their low degree of C-terminal tail sequence similarity with BILF1.

A point mutation that disrupts BILF1 PM GPCR signaling did not abrogate MAVS dislocation, suggesting that BILF1 GPCR signaling and MAVS subversion are genetically separable. How BILF1 distributes to PM versus mitochondrial membrane sites remains unknown. Upon binding to kynurenic acid, host GPCR GPR35 traffics from the PM to the outer mitochondria membrane.^96^ Likewise, Rab5a, which partially co-localized with BILF1, translocates from early endosomes to mitochondrial membranes with oxidative stress.^97^

The signal transduction mediator 14-3-3ε is UFMylated in response to RNA virus infection, which supports RIG-I/MAVS association and their downstream activation of type I interferon responses.^98^ UFL1 is recruited to mitochondrial-associated ER membrane (MAM) upon RNA viral infection.^98,99^ Therefore, although not necessarily required for UFL1 recruitment MAM, we speculate that the BILF1 C-terminal tail evolved the ability to also recruit UFL1 to coordinate MAVS UFMylation. Thus, EBV converted a key antiviral UFMylation signal to subvert MAVS-driven NLRP3 inflammasomes.

We suggest that BILF1 exploits an existing pathway, whereby UFMylation triggers the dislocation of mitochondrial outer membrane proteins to MDVs for lysosomal delivery. MDVs can act as mitochondrial stress responses, functioning prior to or in parallel with mitophagy.^77–80^ Although PARK2 poly-ubiquitination of mitofusins and voltage-dependent anion channels triggers autophagy,^100^ UFMylation may instead trigger selective removal of mitochondrial membrane proteins. Similarly, UFMylation of polypeptides stalled in the endoplasmic reticulum translocon supports their lysosomal degradation.^101^ PARK2 was essential for MAVS MDV packaging, suggesting that UFMylation and poly-ubiquitin chains may together guide its selective mitochondrial extraction and lysosomal delivery. Recently, UFMylation was implicated in CMV targeting of HLA molecules for proteasomal degradation.^102^ However, UFMylation was not detectable on HLA, suggesting a distinct mechanism.

GPCRs are targets of ~ 30% of FDA-approved drugs.^103^ The above studies highlight BILF1 as an intriguing therapeutic target. Antagonists of BILF1 association with MAVS or UFL1 or a small molecule BILF1 degrader compound, promises to re-sensitize cells undergoing lytic replication to pyroptosis. Furthermore, if used together with lytic reactivation strategies, BILF1 antagonists could trigger tumor cell death, although also inducing pro-inflammatory IL-1 and IL-18 responses to induce cellular anti-tumor immune responses.

In summary, a B cell protein EBV lytic cycle protein interaction map revealed a wide array of virus/host interactions. Cross-comparison with HCMV and KSHV proteomic datasets highlighted common and unique herpesvirus host targets. The EBV GPCR BILF1 utilizes its C-terminal tail to trigger MAVS K461 UFMylation, resulting in selective MAVS removal from mitochondrial membranes and routing via MDVs to lysosomes to prevent EBV activation of the NLRP3 inflammasome, which otherwise triggers pyroptosis and limits viral replication.

### Limitations of the study

Our proteomic map used ORFs encoded by type I EBV, as it is more prevalent worldwide and is more transforming. However, a subset of results could differ with type II EBV strain ORFs. We used P3HR-1 cells with type II EBV, although a substantial portion of type I and II polymorphism resides within EBV latency proteins. Likewise, our proteomic studies were performed in B cells, and particular results may differ in epithelial cells. We used doxycycline to induce EBV ORFs, which can cause toxicity over long durations. However, we exposed cells for 15 h to minimize this concern. Attempts to remove sticky proteins by stringent filtering may have created false negative results when hosts or viral proteins truly interacted with multiple EBV baits. With regards to BILF1, studies were conducted in cell line models, since robust primary B cell systems for EBV lytic reactivation are not currently available. Key BILF1 results remain to be validated in EBV+ memory B cells triggered for lytic replication, a model for which does not presently exist.

## STAR★METHODS

### RESOURCE AVAILABILITY

#### Lead contact

Further information and requests for resources and reagents should be directed to and will be fulfilled by the lead contact, Benjamin E. Gewurz (bgewurz@bwh.harvard.edu).

#### Materials availability

All reagents will be made available on request after completion of a Materials Transfer Agreement.

#### Data and code availability

- The mass spectrometry proteomics data have been deposited to the ProteomeXchange Consortium via the PRIDE^104^ partner repository (PRIDE: PXD041336) and made publicly available upon publication. All other raw data have been deposited at Mendeley and are publicly available as of the date of publication (Mendeley Data: https://doi.org/10.17632/zk884935vw.1). Microcopy data reported in this paper will be shared by the lead contact upon request. Figures were drawn with commercially available GraphPad, Biorender and Microsoft Powerpoint.
- This paper does not report original code.
- Any additional information required to reanalyze the data reported in this paper is available from the lead contact upon request.

### EXPERIMENTAL MODEL AND SUBJECT DETAILS

#### Cell Lines

HEK293T were cultured in DMEM supplemented with 10% FBS and 1% Pen/Strep. AGSiZ were cultured in F-12-Glutomax supplemented with 10% FBS, 1% Pen/Strep, 0.5 μg/ml puromycin and 0.5 μg/ml G418. P3HR-1-ZHT/RHT-Cas9+, EBV+ Akata-Cas9+, Daudi-Cas9+ cells and P3HR-1-TMEM192 cells were cultured in RPMI-1640 supplemented with 10% v/v FBS and 1% Pen/Strep. Cas9+ cells were maintained in 5 μg/ml blasticidin. P3HR-1-Z/R-HT were also maintained with 25 μg/ml G418 and 25 μg/ml hygromycin. All cells were incubated at 37°C with 5% CO_2_ and were routinely confirmed to be mycoplasma-negative. Cell lines were authenticated by STIR profiling. cDNA used in this study were cloned into the pLIX-402 vector. pLIX-402 uses a Tet ON TRE promoter to drive expression of the gene of interest with a C-terminal HA Tag. Stable cell lines were generated by lentiviral transduction and antibiotic selection with puromycin (pLIX-402). Cell lines were then maintained with 3.3 μg/ml puromycin.

### METHOD DETAILS

#### Molecular cloning

Unless otherwise specified, all cloning experiments were performed by Gateway recombination. Briefly, 150 ng of the destination vector and donor vector containing the gene of interest were co-incubated with 1X LR Clonase Enzyme Mix (Invitrogen #11789–020) overnight at room temperature. The reaction mixture was then transformed into 50 μl of Stbl3 bacteria, spread on LB plates with corresponding antibiotics. MAVS and BILF1 mutants are generated either by standard restriction-ligation procedures or site-directed mutagenesis according to manufacturer’s protocol (NEB # E0554). Oligos used for mutagenesis are listed in Table S6.

#### Chemical compounds

Unless otherwise specified, EBV lytic induction in P3HR-1, Akata and AGSiZ cells were induced with 400 nM 4-Hydroxytamoxifen (4HT) (Sigma Aldrich) for 24h, 15 μg/ml anti-IgG crosslinking (Agilent) for 48h and 5 μg/ml doxycycline (Sigma Aldrich) for 24h, respectively. Inhibition of NLRP3 inflammasome in lytic-induced BILF1-depleted P3HR-1 cells was achieved by the addition of 10 μM MCC950 (InvivoGen) simultaneously with the EBV lytic inducer 4HT for 24h. NLRP3 inflammasome activation was induced by the potassium ionophore, Nigericin (InvivoGen) at 10 μM for 4h.

#### Sample preparation for LC/MS proteomics analysis

Samples were generated and analyzed in technical duplicate, using the method originally described in Huttlin et al.^32,105^ Whole cell lysates were prepared from 400 million P3HR-1 ZHT/RHT cells per replicate that were induced into the lytic cycle by 4HT (400 nM) and NaB (500 μM) for 24h, and that were also induced to express a HA-tagged bait by doxycycline (5 μg/ml) addition for the final 15hrs. Samples were prepared as previously described.^28^ Briefly, cells were lysed in (50 mM Tris-HCI pH 7.5, 300 mM NaCl, 0.5% v/v NP40, 1 mM DTT and Roche protease inhibitor cocktail). Samples were tumbled for 15 mins at 4°C and subjected to centrifugation at 16,000xg for 15 mins at 4°C. Lysates were then filtered through a 0.7 μm filter and incubated for 3h with immobilized mouse monoclonal anti-HA agarose resin (Sigma). Duplicates samples were combined and washed seven times with lysis buffer, followed by seven PBS washes. Immunopurified proteins were then eluted by addition of 200 μl of 250 μg/ml HA peptide in PBS at 37°C for 30 mins with agitation, followed by another identical peptide elution. Eluted material were then precipitated with 20% trichloroacetic acid (TCA), washed once with 10% TCA, washed three times with cold acetone and dried to completion, using a centrifugal evaporator. Samples were resuspended in digestion buffer (50 mM Tris-HCl pH 8.5, 10% acetonitrile (AcN), 1 mM DTT, 10 μg/ml trypsin (Promega) and incubated overnight at 37°C, with agitation. The reaction was quenched by 50% formic acid (FA), subjected to C18 solid-phase extraction, and vacuum-centrifuged to complete dryness. Samples were reconstituted in 4% AcN/5% FA and divided into technical duplicates prior to LC-MS/MS on an Orbitrap Lumos.

#### LC-MS Proteomic Analysis

Peptides for each sample were analyzed in technical duplicate, with the run order reversed from one batch of replicate analyses to the next to ensure that any carry-over was different in each case. Two washes were used between each sample to further minimize carry-over. Mass spectrometry data were acquired using an Orbitrap Fusion Lumos. An Ultimate 3000 RSLC nano UHPLC equipped with a 300 mm ID × 5 mm Acclaim PepMap m-Precolumn (Thermo Fisher Scientific) and a 75 μm ID × 75 cm 2 μm particle Acclaim PepMap RSLC analytical column was used. Loading solvent was 0.1% v/v FA, and the analytical solvents were (A) 0.1% v/v FA and (B) 80% v/v AcN + 0.1% v/v FA. All separations were carried out at 55C. Samples were loaded at 5 μl/min for 5 min in loading solvent before beginning the analytical gradient. The following gradient was used: 3–7% B over 3 min then 7–37% B over 54 min followed by a 4 min wash in 95% B and equilibration in 3% B for 15 min. The following settings were used: MS1, 350–1500 Thompsons (Th), 120,000 resolution, 2 × 10^5^ automatic gain control (AGC) target, 50 ms maximum injection time. MS2, quadrupole isolation at an isolation width of m/z 0.7, higher-energy collisional dissociation (HCD) fragmentation (normalized collision energy (NCE) 34) with fragment ions scanning in the ion trap from m/z 120, 1 × 10^4^ AGC target, 250 ms maximum injection time, with ions accumulated for all parallelizable times. The method excluded undetermined and very high charge states (≥25+). Dynamic exclusion was set to + /− 10 ppm for 25 s. MS2 fragmentation was triggered on precursors 5 × 10^3^ counts and above. Two 45 min washes were included between every affinity purification-mass spectrometry (AP-MS) analysis, to minimize carry-over between samples. 1 μl transport solution (0.1% v/v trifluoroacetic acid) was injected, over the following gradient: 3–40% B over 29 min followed by a 3 min wash at 95% B and equilibration at 3% B for 10 min.

#### CompPASS identification of high confidence protein interactors

To identify interactors for each bait, replicate pairs were combined to attain a summary of proteins identified in both runs. Data reported for each protein in every IP in the dataset include: (a) the number of peptide spectrum matches (PSMs) averaged between technical replicates; (b) an entropy score, which compares the number of PSM between replicates to eliminate proteins that are not detected consistently; (c) a z-score, calculated in comparison to the average and standard deviation of PSMs observed across all IPs; and (d) an NWD score, which reflects (i) how frequently this protein was detected and (ii) whether it was detected reproducibly. NWD scores were calculated as described in^34^ using the fraction of runs in which a protein was observed, the observed number of PSMs, the average and standard deviation of PSMs observed for that protein across all IPs, and the number of replicates (1 or 2) containing the protein of interest. Protein interactors identified were filtered as described in the legend to Figure 1A. Specifically, stringent filters were applied to remove inconsistent and low-confidence protein identifications across all IPs and thus minimize both false protein identifications and associations.^32^ These included a minimum PSM score of 1.5 (i.e. ≥3 peptides per protein across both replicates) and an entropy score of ≥0.75. Very high confidence interacting proteins (VHCIPs) were defined as prey meeting PSM and entropy criteria with either an NWD≥1.0 or z-score≥4.0, and high confidence interacting proteins (HCIPs) were defined as prey meeting PSM and entropy criteria with either an NWD≥1.0 or z-score≥3.0.

NWD scores were normalized so that the top 2% earned scores of ≥ 1.0. Stringent filters were applied to remove inconsistent and low-confidence protein identifications across all IPs and thus minimize both false protein identifications and associations.^32^ These included a minimum PSM score of 1.5 (i.e. R3 peptides per protein across both replicates) and an entropy score of ≥0.75. Very high confidence interacting proteins (VHCIPs) were defined as prey meeting PSM and entropy criteria with either an NWD≥1.0 or z-scoreR4.0, and high confidence interacting proteins (HCIPs) were defined as prey meeting PSM and entropy criteria with either an NWD≥1.0 or z-score≥3.0 (Table S2). To facilitate global analysis of all data, HCIPs as opposed to VHCIPs were examined for the remainder of this study. Previous studies have estimated a 5% false discovery rate when employing a similar strategy with a top NWD score cutoff of 2%.^33^

#### CRIPSR analysis

CRISPR/Cas9 editing was performed as described.^106^ Briefly, sgRNAs were cloned into pLentiGuide-puro (Addgene plasmid #52963,^107^ pLenti-spBsmBI-sgRNA-Hygro (Addgene plasmid #62205^108^ or LentiGuide-zeo (Addgene plasmid #160091^109^ by restriction-ligation, and sequenced verified. Lentiviral transduction in 293T cells were performed as described previously.^31^ In brief, 293T cells were co-transfected with 500ng lentiviral plasmid, 400ng psPAX2 (a gift from Didier Trono, Addgene plasmid #12260) and 150ng VSV-G plasmids for packaging. Lentivirus produced were filtered with 0.45μm filter and transduced into P3HR-1-Z/R-HT-Cas9+ and Akata-EBV-Cas9+ cells. Transduced cells were selected for 1 week with 0.5 μg/ml puromycin or 2 weeks with 25 μg/ml hygromycin or 100 μg/ml zeocin. CRISPR KOs were verified by western blot analysis or amplicon sequencing (only when antibody is unavailable). sgRNA against genes used in this study are listed in Table S6.

#### Quantification of EBV copy number

Intracellular EBV genome copy # were quantified by qPCR analysis. For intracellular viral DNA extraction, total DNA from 1×10^6^ cells were extracted by the Blood & Cell culture DNA mini kit (Qiagen). Extracted DNA were diluted to 10 ng/μl and were subjected to qPCR targeting the BALF5 gene. Serial dilutions of pHAGE-BALF5 plasmid at 25 ng/μl were used to generate the standard curve. Viral DNA copy number was calculated by substituting sample Cq values into the regression equation dictated by the standard curve. qPCR primer sequences are listed in Table S6.

#### Immunoblot analysis

Immunoblot analyses were performed as described previously.^26^ In brief, whole cell lysates were separated by SDS-PAGE electrophoresis, transferred onto nitrocellulose membrane, blocked with 5% milk in TBST buffer for 1h and incubated with the corresponding primary antibodies at 4°C overnight. Blots were washed 3 times in TBST solution and were incubated with secondary antibodies for 1h at room temperature. Blots were then washed 3 times in TBST solution and were developed by incubating with ECL chemiluminescence. Images were captured by Licor Fc platform. All antibodies used in this study are listed in the key resources table.

#### Flow cytometry analysis

Cells were washed once with cold PBS supplemented with 2% v/v fetal bovine serum (FBS). Cells were then incubated with Cy5-conjugaged anti-gp350 antibody (1:1000) in 2% FBS v/v, PBS for 30 mins at 4°C. Cells were pelleted, washed twice, resuspended in 2% FBS v/v, PBS into flow cytometry-compatible tubes and processed immediately. Flow cytometric data was acquired with a BD FACSCalibur instrument and analysis was performed with FlowJo V10.

#### Immunofluorescence analysis

Cells dried on glass slides were fixed with 4% paraformaldehyde/PBS solution for 10 mins, permeabilized with 0.5% Triton X-100/PBS for 5 mins and blocked with 1% BSA/PBS for 1h at room temperature. Subsequently, cells were incubated with a cocktail of primary antibodies at 1:100 against NLRP3, ASC, TOMM20, MAVS, LAMP1, AIM2, Rab5A, Rab7, Rab27b and LC3B in blocking solution for 1h at 37°C. Cells were then washed twice with PBS and incubated with a cocktail of secondary antibodies at 1:1000 in PBS for 1h at 37°C in the dark. Finally, cells were washed twice with PBS and were stained/mounted overnight with ProLong^™^ Gold Antifade Mountant with DAPI. Image acquisition and analysis was performed with Zeiss LSM 800 instrument and with Zeiss Zen Lite (Blue) software, respectively. Arivis Vision4D from ZEISS ZEN lite (blue edition) was used for 3D reconstruction in Figures S5 and S6. Image J was used to score the % of colocalization of MAVS-TOMM20, NLRP3-ASC, NLRP3-AIM2 and MAVS-LAMP1 in P3HR-1 cells, using the ImageJ “ComDet” plugin.

#### Live cell imaging

MAVS cDNA (DNASU#HsCD00296475) was cloned into pDEST-CMV-N-EGFP plasmid (a gift from Robin Ketteler, Addgene plasmid #122842^110^ by Gateway cloning to produce pDEST-CMV-N-EGFP-MAVS plasmid. To visualize the mitochondria, the mito-BFP plasmid (a gift from Gia Voeltz, Addgene plasmid #49151^111^ was used. Mito-BFP expresses BFP fused to an N-terminal mitochondrial targeting signal sequence obtained from COX4 amino acids 1–21. AGSiZ cells were plated in glass bottom 35 mm dish (ibidi #81158) at 0.4 million/ml. The next day, the plated cells were replenished with fresh medium at least 1h prior to co-transfection by pDEST-CMV-N-EGFP-MAVS and mito-BFP plasmids using Lipofectamine 2000 (Thermo) according to manufacturer’s instruction. Transfection medium was replaced by fresh medium after 6h and cells were incubated for an addition of 18h before live cell imaging. EBV lytic cycle was induced with 10 μg/ml Doxycycline and active lysosome was stained by 200 nM SiR-lysosome (#CY-SC012, Cytoskeleton, Inc.) during the course of experiment. At 200 mins post-lytic induction, image acquisition was performed with Zeiss LSM 800 instrument at an interval of 10 s for 15 mins and Zeiss Zen Lite (Blue) software was used for image analysis.

#### Co-immunoprecipitation analysis

Expression of either BILF1-WT, BILF1-ΔN or BILF1-ΔC were induced by the addition of 5 mg/ml doxycycline, 5 nM of Bortezomib was added to preserve UFMylation and 400 nM 4HT was used to induce the lytic cycle in P3HR-1 cells. 100 million cells were harvested and was lysed in ice cold lysis buffer (1% v/v NP40, 150 mM Tris, 300 mM NaCl in dH2O) supplemented with 1X cOmplete^™^ EDTA-free protease inhibitor cocktail (Sigma), 1 mM Na3VO4 and 1 mM NaF for 1h at 4°C with rotation. Lysed cells were pelleted, and lysates were incubated with anti-HA tag magnetic beads (Pierce, Thermo) at 4°C overnight. Beads were washed with lysis buffer for four times and were eluted using 1X SDS loading buffer incubated for 10 mins at 95°C. Proteins were separated by SDS-PAGE gel and transferred to nitrocellulose membranes. Subsequent procedures were similar to that mentioned in “Immunoblot analysis”

#### UFMylation co-immunoprecipitation analysis

Expression of MAVS was induced by addition of 5 μg/ml doxycycline. 100 million cells were harvested and lysed in ice cold lysis buffer (1% v/v NP40, 50 mM Tris, 150 mM NaCl, 0.5% w/v sodium deoxycholate, 10% glycerol in dH2O) supplemented with 1X cOmplete^™^ EDTA-free protease inhibitor cocktail (Sigma), 1 mM Na3VO4, 1 mM NaF and 20 mM N-Ethylmaleimide (NEM, Sigma-Aldrich) for 1h at 4°C with rotation. Lysed cells were pelleted, and lysates were boiled at 100°C for 5 mins and then re-pelleted. Lysates were then precleared with 0.2 μg anti-mouse IgG isotype antibodies and protein A/G magnetic beads (Pierce, Thermo) for 30 mins at 4°C with rotation. Precleared lysates were then incubated with anti-MAVS antibody for 1 h at 4°C. Protein A/G magnetic beads were then added to the immunocomplex and were incubated overnight at 4°C. Beads were washed with cold lysis-boil buffer for four times and were eluted using 1X SDS loading buffer incubated for 10 mins at 95°C. Proteins were separated by SDS-PAGE gel and transferred to nitrocellulose membranes. Subsequent procedures were similar to that mentioned in “Immunoblot analysis”

#### Sample preparation and LC-MS/MS for post-translational modification analysis

Samples were reduced, alkylated and digested in-gel using trypsin. The resulting peptides were dried and re-suspended 20 μl 3% MeCN/0.1% TFA.

Mass spectrometry data was acquired using a Q Exactive Plus coupled to an Ultimate 3000 RSLC nano UHPLC equipped with a 100 μm ID × 2 cm Acclaim PepMap Precolumn (Thermo Fisher Scientific) and a 50 μm ID × 50 cm, 2 μm particle Acclaim PepMap RSLC analytical column. Loading solvent was 0.1% FA with analytical solvents A: 0.1% FA and B: 80% MeCN + 0.1% FA. Samples were loaded at 5 μl/minute loading solvent for 5 minutes before beginning the analytical gradient. The analytical gradient was 10–40% B over 42 minutes rising to 95% B by 45 minutes followed by a 4 minute wash at 95% B and equilibration at 3% solvent B. Columns were held at 40°C. Data was acquired in a DDA fashion with the following settings: MS1: 400–1500 Th, 70,000 resolution, 1×10^5^ AGC target, 250 ms maximum injection time. MS2: Quadrupole isolation at an isolation width of m/z 3.0, HCD fragmentation (NCE 30). Dynamic exclusion was set for 30 s. MS2 fragmentation was trigged on precursors 3.2 ×10^4^ counts and above.

Raw files were processed using PEAKS X Pro (Bioinformatics Solutions Inc.). Data was searched against a human Uniprot Database (downloaded 12/1/21) and a database of common contaminants. The data was searched using the following variable PTMs: carbamidomethylation on cysteine residues, oxidation on methionine residues and UFMylation on lysine residues. In Figure *, peptides identified by de novo sequencing in PEAKS software are not shown.

#### Lysosome-immunoprecipitation and mitochondria-immunoprecipitation

Lysosome-immunoprecipitation or mitochondria-immunoprecipitation was performed as described previously.^112,113^ In brief, 35 million P3HR-1- stably expressing HA-TMEM192 or HA-OMP25 cells were harvested and washed twice with cold PBS. The cells were resuspended in 1 ml of cold KPBS (136 mM KCl, 10 mM KH2PO4, pH7.25) and were then centrifuged at 1000 xg for 2 mins at 4°C. Pelleted cells were resuspended in 500 μl of ice cold KPBS and were homogenized on ice with a 1 ml homogenizer. 2.5% of the homogenate was reserved as input and the remaining fraction was centrifuged at max speed for 15 mins at 4°C. The lysosome or mitochondria containing supernatant fraction was then incubated with 150 μl of anti-HA magnetic beads (Pierce, Thermo) prewashed with KPBS for 15 mins at 4°C with rotation. Beads were washed with cold KPBS for three times and were eluted using 1X SDS loading buffer incubated for 10 mins at 95°C. Subsequent procedures were similar to that mentioned in “Immunoblot analysis”

#### Caspase-1 activity measurement

Caspase-1 activity in this study was measured using Caspase-Glo^®^ 1 Inflammasome Assay from Promega (#G9951) according to manufacturer’s instructions. In brief, cells were mixed with Caspase-Glo^®^ 1 reagent at a 1:1 ratio and incubated for 1h at room temperature. Luminescence signal was measured using a Molecular Devices microplate reader. Final caspase-1 activities presented were determined by normalizing the signal against live cell number, as determined by trypan blue vital dye exclusion.

#### ASC polymerization

ASS polymerization was performed as described previously. Briefly, 3 million cells were harvested and washed with cold PBS once. The pellet was homogenized with 100 μl 1% NP-40 and was centrifuged at max speed for 5 mins at 4°C. Supernatant was collected, mixed with 4X SDS loading buffer and boiled for 10 mins. 1mM DSS (disuccinimidyl suberate) in PBS was added to the pellet and incubated in shaking incubators for 15 mins at 37°C. DSS solution is removed followed by centrifugation at max speed for 5 mins. The pellet was resuspended in 1X SDS loading buffer and boiled for 10 mins. Subsequent procedures were similar to that mentioned in “Immunoblot analysis”

#### Structural prediction

Structural prediction for BILF1-MAVS and BILF1-UFL1 was performed with AlphaFold Collab- Multimer^73^ and Robetta server,^114^ respectively.Structure visualization was performed with PyMOL version 2.0.

### QUANTIFICATION AND STATISTICAL ANALYSIS

Unless otherwise indicated, all bar and line graphs represent the arithmetic mean of three independent experiments (n = 3), with error bars denoting SEM. Significance between the control and experimental groups, or indicated pairs of groups, was assessed using the unpaired Student’s t-test in the GraphPad Prism 7 software. P values correlate with symbols as follows, unless otherwise indicated: ns = not significant, p > 0.05; *p % 0.05; **p % 0.01; ***p % 0.001; ****p % 0.0001.

#### Network Assembly

To facilitate cross-species comparisons, we combined our EBV interactions with published interaction networks from human Cytomegalovirus and Kaposi’s Sarcoma Associated Herpesvirus,^28,29^ as well as human protein-protein interactions from the BioPlex project.^47^ For this analysis, interactions of human proteins observed in both 293T and HCT116 cell lines were merged. Across all viral interaction datasets, human preys were mapped to Entrez Gene ID’s and then merged with the combined BioPlex network to create a single composite interaction network. Interactions among virus proteins in each species were included as well. Depending on the analysis, EBV interactions were filtered using either stringent or relaxed criteria prior to incorporation.

#### Pathway and cluster analysis

The Database for Annotation, Visualization and Integrated Discovery (DAVID) 2021 version^41^ was used to determine pathway enrichment for Figures 1B and 1C and Table S3. Indicated subsets of human HCIPs were searched against a background of all human proteins, using default settings.

To identify baits that interact with host E1s, E2s, E3s, cullins, deubiquitinases (DUB), proteasome components (PSM) and ubiquitin-binding proteins (UBP), HCIPs were searched against a comprehensive list provided in https://elifesciences.org/articles/40009#s4.^45^ To supplement this list with E3s that ligate other moieties in addition to ubiquitin (such as SUMO, UFM), HCIPs that included the term ‘E3’ in their protein name were additionally searched.

Hierarchical centroid clustering based on uncentered Pearson correlation was performed using Cluster 3.0 (Stanford University) and visualised using Java Treeview (http://jtreeview.sourceforge.net).

#### Proximity Scoring of CORUM Complexes

An approach based on network propagation^115^ was used to quantify the proximity of human proteins and protein complexes to proteins belonging to each virus. Each viral proteome was scored separately via network propagation. To begin, each viral protein was assigned a starting weight of 1.0 with all other proteins assigned a weight of zero. Forty iterations of a Random Walk were then performed while allowing restarts at each step with probability 0.5. The final weights assigned to each protein were then returned. This procedure was then repeated across 100 additional randomized versions of the combined human-virus protein-protein interaction network. Network randomization preserved bait and prey identities as described previously.^47^

To identify protein complexes that were associated with each virus, “core” complexes from the CORUM database^48^ were downloaded and scored individually. To score a complex, its constituent proteins were identified in the network and their network propagation scores were extracted and summed. Any complex members not found in the interaction network were ignored in this analysis. This process was then repeated across all 100 randomized networks and the resulting scores were used to determine averages and standard deviations that could be used to convert the complex’s summed propagation score into a Z-score. To consider a complex associated with a particular virus, we required a minimum Z-score of 4 and a minimum summed propagation score of 0.04. Identical criteria were used for all three viruses and were found to correspond to approximately a 5% FDR based on scoring of each virus against an additional randomized network. Scores are provided in Table S4.

For purposes of plotting (see Figure 2B), CORUM complexes were displayed if their Z-scores observed across all three cell lines summed to 20 or greater. This was simply done to ensure that the resulting figure was legible. Plots of individual complexes (Figures 2C and 2D) were generated by extracting CORUM complex members and all viral proteins within a graph distance of 2 or less of at least one complex member. Network layouts were calculated via gravity embedding, and unconnected nodes were manually removed.

Network analyses and plotting were performed using *Mathematica 13.1* (Wolfram Research).

#### Interaction Network Web Browser

A custom viewer for our combined viral-human protein interaction network is available at https://wren.hms.harvard.edu/ViroPlex/. It was implemented using R^116^ as well as R-Shiny.^117^ Our viewer includes interactions for all three viral interactomes – including both stringent and relaxed filtering of EBV interactions – as well as human protein-protein interactions drawn from BioPlex. To improve performance, only those human protein-protein interactions within max distance of 2 are displayed in the viewer.

## Supplementary Material

Table S3

Table S4

Table S5

Table S6

Figures S1–S6 and supplemental reference

Table S1

Movie S1

Table S2

## Figures and Tables

**KEY RESOURCES TABLE:** 

REAGENT or RESOURCE	SOURCE	IDENTIFIER

Antibodies		

Anti-EBV ZEBRA mouse monoclonal antibody (BZ1)	Santa Cruz Biotechnology	cat# sc-53904; RRID: AB_783257
Anti-EBV Ea-D mouse monoclonal antibody (0261)	Santa Cruz Biotechnology	cat# sc-58121; RRID: AB_631448
Anti-gp350/220 mouse monoclonal antibody (OT6)	A gift from Jaap M Middeldorp	N/A
Anti-EBV BHRF1 rabbit polyclonal antibody	Thermo Fisher Scientific	cat# PA5-117549; RRID: AB_2902178
Anti-GAPDH XP^®^ rabbit monoclonal antibody (D16H11)	Cell Signaling Technology	cat# 5174; RRID: AB_10622025
Anti-HA.11 tag mouse monoclonal antibody (16B12)	Biolegend	cat# 901513; RRID: AB_2820200
Anti-NLRP3 rabbit polyclonal antibody	Proteintech	cat# 19771-1-AP; RRID: AB_10646484
Anti-ASC/TMS1 rabbit monoclonal antibody (E1E3I)	Cell Signaling Technology	cat# 13833; RRID: AB_2798325
Anti- ASC/TMS1 mouse monoclonal antibody	Proteintech	cat# 67494-1-Ig; RRID: AB_2882718
Anti-AIM2 mouse monoclonal antibody	Biolegend	cat# 652802; RRID: AB_2561540
Anti-TOMM20 mouse monoclonal antibody (F-10)	Santa Cruz Biotechnology	cat# sc-17764; RRID: AB_628381
Anti-MAVS rabbit polyclonal antibody	Proteintech	cat# 14341-1-AP; RRID: AB_10548408
Anti-MAVS mouse monoclonal antibody	Proteintech	cat# 66911-1-Ig; RRID: AB_2882238
Anti-MAVS rabbit polyclonal antibody (Position: L34-Q96)	Thermo Fisher Scientific	cat# PA5-79636; RRID: AB_2746751
Anti-RIG-I rabbit monoclonal antibody (D14G6)	Cell Signaling Technology	cat# 3743; RRID: AB_2269233
Anti-GFP rabbit polyclonal antibody	Proteintech	cat# 50430-2-AP; RRID: AB_11042881
Anti- mCherry rabbit monoclonal antibody (E5D8F)	Cell Signaling Technology	cat# 43590; RRID: AB_2799246
Anti-UFM1 rabbit polyclonal antibody	Proteintech	cat# 15883-1-AP; RRID: AB_2878195
Anti-UFL1 rabbit polyclonal antibody	Proteintech	cat# 26087-1-AP; RRID: AB_2880370
Anti-UFC1 rabbit polyclonal antibody	Proteintech	cat# 15783-1-AP; RRID: AB_2213938
Anti-IL-1β rabbit polyclonal antibody	Novus Biologicals	cat# NB600633; RRID: AB_577977
Anti-GSDMD rabbit polyclonal antibody	Abcam	cat# ab155233; RRID: AB_2736999
Anti-LAMP1 mouse monoclonal antibody (D4O1S)	Cell Signaling Technology	cat# 15665; RRID: AB_2798750
Anti-PMP70 (ABCD3) mouse monoclonal antibody	Proteintech	cat# 66697-1-Ig; RRID: AB_2882050
Anti-Rab5A mouse monoclonal antibody (E6N8S)	Cell Signaling Technology	cat# 46449; RRID: AB_2799303
Anti-Rab7 mouse monoclonal antibody (E9O7E)	Cell Signaling Technology	cat# 95746; RRID: AB_2800252
Anti-LC3B mouse monoclonal antibody (E5Q2K)	Cell Signaling Technology	cat# 83506; RRID: AB_2800018
Anti-Rab27b mouse monoclonal antibody	Proteintech	cat# 66944-1-Ig; RRID: AB_2882268
Anti-α-tubulin rabbit monoclonal antibody (11H10)	Cell Signaling Technology	cat# 2125; RRID: AB_2619646
Anti-PARK2 mouse monoclonal antibody (Prk8)	Cell Signaling Technology	cat# 4211; RRID: AB_2159920
Anti-Drp1 rabbit polyclonal antibody (N-terminal)	Proteintech	cat# 26187-1-AP; RRID: AB_2880417
Anti-CNOT1 rabbit polyclonal antibody	Proteintech	cat# 14276-1-AP; RRID: AB_10888627
Anti-CNOT10 rabbit polyclonal antibody	Proteintech	cat# 15938-1-AP; RRID: AB_2229678
Anti-IRF3 rabbit polyclonal antibody	Proteintech	cat# 11312-1-AP; RRID: AB_2127004
Anti-dsRNA mouse monoclonal antibody (clone rJ2)	Miilipore-Sigma	cat# MABE1134; RRID: AB_2819101
Anti-Mouse IgG HRP-coupled secondary antibody	Cell Signaling Technology	cat# 7076; RRID: AB_330924
Anti-Rabbit IgG HRP-coupled secondary antibody	Cell Signaling Technology	cat# 7074; RRID: AB_2099233
Rabbit anti-Mouse IgG (H+L) Cross-Adsorbed Secondary Antibody, Alexa Fluor 647	Thermo Fisher Scientific	cat# A-21239; RRID: AB_2535808
Goat anti-Mouse IgG (H+L) Cross-Adsorbed Secondary Antibody, Alexa Fluor 488	Thermo Fisher Scientific	cat# A-11001; RRID: AB_143160
Goat anti-Rabbit IgG (H+L) Cross-Adsorbed Secondary Antibody, Alexa Fluor 647	Thermo Fisher Scientific	cat# A-21244; RRID: AB_2535812
Goat anti-Rabbit IgG (H+L) Cross-Adsorbed Secondary Antibody, Alexa Fluor 488	Thermo Fisher Scientific	cat# A-11008; RRID: AB_143165
Anti-Human IgG rabbit polyclonal antibody (Gamma-Chains)	Agilent	cat# A042402; RRID: AB_578517

Chemicals, peptides, and recombinant proteins		

Pierce^™^ Protein A/G Magnetic Beads	Thermo Fisher Scientific	88803
Pierce^™^ Anti-HA Magnetic Beads	Thermo Fisher Scientific	88837
T4 DNA ligase	New England Biolabs	M0202L
Doxycycline hyclate	Sigma-Aldrich	D9891-1G
(Z)-4-Hydroxytamoxifen	Sigma-Aldrich	H7904-25MG
NAE Inhibitor, MLN4924	Sigma-Aldrich	5.05477
Bortezomib (PS-341)	APExBIO	A2614
Sir-Lysosome Kit	Cytoskeleton Inc.	CY-SC012
NLRP3 inhibitor, MCC950	A gift from Jonathan Kagan; InvivoGen	MCC950
Caspase-1/4 and Pyroptosis inhibitor, VX765	InvivoGen	VX765
Nigericin	InvivoGen	tlrl-nig
Lipofectamine^™^ 2000 Transfection Reagent	Thermo Fisher Scientific	11668019
Sodium butyrate, >98%, Alfa Aesar^™^	Thermo Fisher Scientific	AAA1107922
2-NBDG (2-(N-(7-Nitrobenz-2-oxa-1,3-diazol-4-yl)Amino)-2-Deoxyglucose)	Thermo Fisher Scientific	N13195
NP40	Sigma-Aldrich	74385-1L
Sequencing Grade Modified Trypsin (Mass Spec Grade) (lyophilized)	Promega	V5111-5x20μg
HA Synthetic Peptide	Thermo Fisher Scientific	26184-5mg
Monoclonal Anti-HA–Agarose antibody produced in mouse	Sigma-Aldrich	A2095-1ML
Formaldehyde solution	Sigma-Aldrich	F8775
cOmplete^™^, Mini, EDTA-free Protease Inhibitor Cocktail	Roche	11697498001
N-Ethylmaleimide (NEM)	Sigma-Aldrich	E3876
Puromycin Dihydrochloride	Thermo Fisher Scientific	A1113803
Hygromycin B	Millipore	400052
G418 Sulfate Solution (50 mg/mL)	GeminiBio	400113
Blasticidin	InvivoGen	ant-bl-5
Zeocin^™^ Selection Reagent	Thermo Fisher Scientific	R25001
TransIT^®^-LT1 Transfection Reagent	Mirus Bio	MIR 2306
DSS (disuccinimidyl suberate)	Thermo Fisher Scientific	21555

Critical commercial assays		

RNeasy Mini Kit	Qiagen	74104
QiAquick PCR Purification Kit	Qiagen	28106
QIAprep Spin Miniprep Kit	Qiagen	27106
DNeasy Blood& Tissue Kit	Qiagen	69504
QIAquick Gel Extraction Kit	Qiagen	28704
Power SYBR Green PCR Master Mix	Applied Biosystems	4367659
Gateway^™^ LR Clonase^™^ II Enzyme Mix	Invitrogen	11789-020
Caspase-Glo^®^ 1 Inflammasome Assay	Promega	G9951
Dual-Luciferase^®^ Reporter Assay System	Promega	E1910

Deposited data		

Mendeley dataset	https://doi.org/10.17632/zk884935vw.1	
Mass spectrometry raw files and associated unmodified peptide and protein quantitation data	Data are available via ProteomeXchange PRIDE: PXD041336.^104^	

Experimental models: Cell lines		

EBV+ Burkitt lymphoma P3HR-1 ZHT	A gift from Eric Johannsen	N/A
EBV+ Burkitt lymphoma AKATA-Cas9	Guo et al.^106^	N/A
EBV+ Burkitt lymphoma Daudi-Cas9	Ma et al., 2017	N/A
HEK293T	ATCC	CRL-3216
EBV+ Gastric carcinoma AGSiZ	A gift from Sankar Swaminathan	N/A
EBV+ Burkitt lymphoma P3HR-1 ZHT-TMEM192	This study	N/A
EBV+ Burkitt lymphoma P3HR-1 ZHT-OMP25	This study	N/A
HEK293T sgUFL1	This study	N/A
EBV+ Burkitt lymphoma P3HR-1 ZHT sgBILF1	This study	N/A
EBV+ Burkitt lymphoma P3HR-1 ZHT sgBXL1	This study	N/A
EBV+ Burkitt lymphoma P3HR-1 ZHT sgBHRF1	This study	N/A
EBV+ Burkitt lymphoma P3HR-1 ZHT sgBILF1/MAVS	This study	N/A
EBV+ Burkitt lymphoma P3HR-1 ZHT sgBILF1/RIG-I	This study	N/A
EBV+ Burkitt lymphoma P3HR-1 ZHT sgPARK2	This study	N/A
EBV+ Burkitt lymphoma P3HR-1 ZHT sgDrp1	This study	N/A
EBV+ Burkitt lymphoma P3HR-1 ZHT sgUFL1	This study	N/A
EBV+ Burkitt lymphoma P3HR-1 ZHT sgUFC1	This study	N/A
EBV+ Burkitt lymphoma P3HR-1 ZHT pLIX402-Zta	This study	N/A
EBV+ Burkitt lymphoma P3HR-1 ZHT pLIX402-BRLF1	This study	N/A
EBV+ Burkitt lymphoma P3HR-1 ZHT pLIX402-BDLF3.5	This study	N/A
EBV+ Burkitt lymphoma P3HR-1 ZHT pLIX402-BALF5	This study	N/A
EBV+ Burkitt lymphoma P3HR-1 ZHT pLIX402-BGLF3.5	This study	N/A
EBV+ Burkitt lymphoma P3HR-1 ZHT pLIX402-BNLF2b	This study	N/A
EBV+ Burkitt lymphoma P3HR-1 ZHT pLIX402-BKRF3	This study	N/A
EBV+ Burkitt lymphoma P3HR-1 ZHT pLIX402-BVLF1.5	This study	N/A
EBV+ Burkitt lymphoma P3HR-1 ZHT pLIX402-BLLF3	This study	N/A
EBV+ Burkitt lymphoma P3HR-1 ZHT pLIX402-BRRF1	This study	N/A
EBV+ Burkitt lymphoma P3HR-1 ZHT pLIX402-BFLF2	This study	N/A
EBV+ Burkitt lymphoma P3HR-1 ZHT pLIX402-BGLF3	This study	N/A
EBV+ Burkitt lymphoma P3HR-1 ZHT pLIX402-LF1	This study	N/A
EBV+ Burkitt lymphoma P3HR-1 ZHT pLIX402-SM	This study	N/A
EBV+ Burkitt lymphoma P3HR-1 ZHT pLIX402-BALF1/0	This study	N/A
EBV+ Burkitt lymphoma P3HR-1 ZHT pLIX402-BFRF1	This study	N/A
EBV+ Burkitt lymphoma P3HR-1 ZHT pLIX402-LF2	This study	N/A
EBV+ Burkitt lymphoma P3HR-1 ZHT pLIX402-BGLF4	This study	N/A
EBV+ Burkitt lymphoma P3HR-1 ZHT pLIX402-BGLF5	This study	N/A
EBV+ Burkitt lymphoma P3HR-1 ZHT pLIX402-BFLF1	This study	N/A
EBV+ Burkitt lymphoma P3HR-1 ZHT pLIX402-BXLF1	This study	N/A
EBV+ Burkitt lymphoma P3HR-1 ZHT pLIX402-BALF3	This study	N/A
EBV+ Burkitt lymphoma P3HR-1 ZHT pLIX402-BBLF2/3	This study	N/A
EBV+ Burkitt lymphoma P3HR-1 ZHT pLIX402-BcRF1	This study	N/A
EBV+ Burkitt lymphoma P3HR-1 ZHT pLIX402-BBLF4	This study	N/A
EBV+ Burkitt lymphoma P3HR-1 ZHT pLIX402-BSLF1	This study	N/A
EBV+ Burkitt lymphoma P3HR-1 ZHT pLIX402-BALF2	This study	N/A
EBV+ Burkitt lymphoma P3HR-1 ZHT pLIX402-BNRF1	This study	N/A
EBV+ Burkitt lymphoma P3HR-1 ZHT pLIX402-BBLF1	This study	N/A
EBV+ Burkitt lymphoma P3HR-1 ZHT pLIX402-BLRF1	This study	N/A
EBV+ Burkitt lymphoma P3HR-1 ZHT pLIX402-BFRF0.5	This study	N/A
EBV+ Burkitt lymphoma P3HR-1 ZHT pLIX402-BKRF2	This study	N/A
EBV+ Burkitt lymphoma P3HR-1 ZHT pLIX402-BLLF2	This study	N/A
EBV+ Burkitt lymphoma P3HR-1 ZHT pLIX402-BLRF2	This study	N/A
EBV+ Burkitt lymphoma P3HR-1 ZHT pLIX402-BCRF1	This study	N/A
EBV+ Burkitt lymphoma P3HR-1 ZHT pLIX402-BFRF3	This study	N/A
EBV+ Burkitt lymphoma P3HR-1 ZHT pLIX402-BKRF4	This study	N/A
EBV+ Burkitt lymphoma P3HR-1 ZHT pLIX402-BSRF1	This study	N/A
EBV+ Burkitt lymphoma P3HR-1 ZHT pLIX402-BZLF2	This study	N/A
EBV+ Burkitt lymphoma P3HR-1 ZHT pLIX402-BDLF3	This study	N/A
EBV+ Burkitt lymphoma P3HR-1 ZHT pLIX402-BILF2	This study	N/A
EBV+ Burkitt lymphoma P3HR-1 ZHT pLIX402-BXRF1	This study	N/A
EBV+ Burkitt lymphoma P3HR-1 ZHT pLIX402-BBRF2	This study	N/A
EBV+ Burkitt lymphoma P3HR-1 ZHT pLIX402-BDLF1	This study	N/A
EBV+ Burkitt lymphoma P3HR-1 ZHT pLIX402-BILF1	This study	N/A
EBV+ Burkitt lymphoma P3HR-1 ZHT pLIX402-BGLF2	This study	N/A
EBV+ Burkitt lymphoma P3HR-1 ZHT pLIX402-BdRF1	This study	N/A
EBV+ Burkitt lymphoma P3HR-1 ZHT pLIX402-BMRF2	This study	N/A
EBV+ Burkitt lymphoma P3HR-1 ZHT pLIX402-BORF1	This study	N/A
EBV+ Burkitt lymphoma P3HR-1 ZHT pLIX402-BTRF1	This study	N/A
EBV+ Burkitt lymphoma P3HR-1 ZHT pLIX402-BBRF3	This study	N/A
EBV+ Burkitt lymphoma P3HR-1 ZHT pLIX402-BDLF2	This study	N/A
EBV+ Burkitt lymphoma P3HR-1 ZHT pLIX402-BGLF1	This study	N/A
EBV+ Burkitt lymphoma P3HR-1 ZHT pLIX402-BRRF2	This study	N/A
EBV+ Burkitt lymphoma P3HR-1 ZHT pLIX402-BVRF1	This study	N/A
EBV+ Burkitt lymphoma P3HR-1 ZHT pLIX402-BVRF2	This study	N/A
EBV+ Burkitt lymphoma P3HR-1 ZHT pLIX402-BBRF1	This study	N/A
EBV+ Burkitt lymphoma P3HR-1 ZHT pLIX402-B-GD-RF1	This study	N/A
EBV+ Burkitt lymphoma P3HR-1 ZHT pLIX402-BXLF2	This study	N/A
EBV+ Burkitt lymphoma P3HR-1 ZHT pLIX402-BALF4	This study	N/A
EBV+ Burkitt lymphoma P3HR-1 ZHT pLIX402-gp350/220	This study	N/A
EBV+ Burkitt lymphoma P3HR-1 ZHT pLIX402-BOLF1	This study	N/A
EBV+ Burkitt lymphoma P3HR-1 ZHT pLIX402-BcLF1	This study	N/A
EBV+ Burkitt lymphoma P3HR-1 ZHT pLIX402-BHRF1	This study	N/A
EBV+ Burkitt lymphoma P3HR-1 ZHT pLIX402-BARF1	This study	N/A
EBV+ Burkitt lymphoma P3HR-1 ZHT pLIX402-BaRF1	This study	N/A
EBV+ Burkitt lymphoma P3HR-1 ZHT pLIX402-BFRF2	This study	N/A
EBV+ Burkitt lymphoma P3HR-1 ZHT pLIX402-BORF2	This study	N/A
EBV+ Burkitt lymphoma P3HR-1 ZHT pLIX402-BPLF1 1–1000	This study	N/A
EBV+ Burkitt lymphoma P3HR-1 ZHT pLIX402-BPLF1 501–1500	This study	N/A
EBV+ Burkitt lymphoma P3HR-1 ZHT pLIX402-BPLF1 1001–2000	This study	N/A
EBV+ Burkitt lymphoma P3HR-1 ZHT pLIX402-BPLF1 1501–2500	This study	N/A
EBV+ Burkitt lymphoma P3HR-1 ZHT pLIX402-BPLF1 2001–3000	This study	N/A
EBV+ Burkitt lymphoma P3HR-1 ZHT pLIX402-BPLF1 3001–3149	This study	N/A
EBV+ Burkitt lymphoma P3HR-1 ZHT pLIX402-BNLF2a	This study	N/A
EBV+ Burkitt lymphoma P3HR-1 ZHT pLIX402-BDLF4	This study	N/A

Oligonucleotides		

CRISPR sgRNAs are listed in Table S6	This paper	N/A
qPCR primers for EBV copy number quantification are listed in Table S6	This paper	N/A
Primers used for amplicon sequencing on *BILF1* gene are listed in Table S6	This paper	N/A
gBlocks and oligos used for molecular cloning of MAVS and BILF1 mutants are listed in Table S6	This paper	N/A

Recombinant DNA		

pLentiGuide-Puro	A gift from Feng Zhang (Addgene plasmid # 52963; http://n2t.net/addgene:52963)^107^	RRID:Addgene_52963
pLenti SpBsmBI sgRNA Hygro	A gift from Rene Maehr (Addgene plasmid # 62205; http://n2t.net/addgene:62205)^108^	RRID:Addgene_62205
LentiGuide-zeo	A gift from Rizwan Haq (Addgene plasmid # 160091; http://n2t.net/addgene:160091)^109^	RRID:Addgene_160091
pLX-TRC313	Broad Institute	N/A
pLX-402	Broad Institute	N/A
pDEST-CMV-N-EGFP	A gift from Robin Ketteler (Addgene plasmid # 122842; http://n2t.net/addgene:122842)^110^	RRID:Addgene_122842
pDEST-CMV-N-mCherry	A gift from Robin Ketteler (Addgene plasmid # 123215; http://n2t.net/addgene:123215)^110^	RRID:Addgene_123215)
pHAGE-C-HA-FLAG	A gift from James Decaprio	N/A
Mito-BFP	A gift from Gia Voeltz (Addgene plasmid # 49151; http://n2t.net/addgene:49151)^111^	RRID:Addgene_49151
pLJC6-3XHA-TMEM192	A gift from David Sabatini (Addgene plasmid # 104434; http://n2t.net/addgene:104434)^112^	RRID:Addgene_104434
pMXs-3XHA-EGFP-OMP25	A gift from David Sabatini (Addgene plasmid # 83356; http://n2t.net/addgene:83356)^113^	RRID:Addgene_83356
pDONR221-MAVS	DNASU	HsCD00296475
pENTR223-UFM1	DNASU	HsCD00504493
pENTR-Zta	A gift from Eric Johannsen	N/A
pENTR-BRLF1	Eric Johannsen	N/A
pENTR-BDLF3.5	Eric Johannsen	N/A
pENTR-BALF5	Eric Johannsen	N/A
pENTR-BGLF3.5	Eric Johannsen	N/A
pENTR-BNLF2b	Eric Johannsen	N/A
pENTR-BKRF3	Eric Johannsen	N/A
pENTR-BVLF1.5	Eric Johannsen	N/A
pENTR-BLLF3	Eric Johannsen	N/A
pENTR-BRRF1	Eric Johannsen	N/A
pENTR-BFLF2	Eric Johannsen	N/A
pENTR-BGLF3	Eric Johannsen	N/A
pENTR-LF1	Eric Johannsen	N/A
pENTR-SM	Eric Johannsen	N/A
pENTR-BALF1/0	Eric Johannsen	N/A
pENTR-BFRF1	Eric Johannsen	N/A
pENTR-LF2	Eric Johannsen	N/A
pENTR-BGLF4	Eric Johannsen	N/A
pENTR-BGLF5	Eric Johannsen	N/A
pENTR-BFLF1	Eric Johannsen	N/A
pENTR-BXLF1	Eric Johannsen	N/A
pENTR-BALF3	Eric Johannsen	N/A
pENTR-BBLF2/3	Eric Johannsen	N/A
pENTR-BcRF1	Eric Johannsen	N/A
pENTR-BBLF4	Eric Johannsen	N/A
pENTR-BSLF1	Eric Johannsen	N/A
pENTR-BALF2	Eric Johannsen	N/A
pENTR-BNRF1	Eric Johannsen	N/A
pENTR-BBLF1	Eric Johannsen	N/A
pENTR-BLRF1	Eric Johannsen	N/A
pENTR-BFRF0.5	Eric Johannsen	N/A
pENTR-BKRF2	Eric Johannsen	N/A
pENTR-BLLF2	Eric Johannsen	N/A
pENTR-BLRF2	Eric Johannsen	N/A
pENTR-BCRF1	Eric Johannsen	N/A
pENTR-BFRF3	Eric Johannsen	N/A
pENTR-BKRF4	Eric Johannsen	N/A
pENTR-BSRF1	Eric Johannsen	N/A
pENTR-BZLF2	Eric Johannsen	N/A
pENTR-BDLF3	Eric Johannsen	N/A
pENTR-BILF2	Eric Johannsen	N/A
pENTR-BXRF1	Eric Johannsen	N/A
pENTR-BBRF2	Eric Johannsen	N/A
pENTR-BDLF1	Eric Johannsen	N/A
pENTR-BILF1	Eric Johannsen	N/A
pENTR-BGLF2	Eric Johannsen	N/A
pENTR-BdRF1	Eric Johannsen	N/A
pENTR-BMRF2	Eric Johannsen	N/A
pENTR-BORF1	Eric Johannsen	N/A
pENTR-BTRF1	Eric Johannsen	N/A
pENTR-BBRF3	Eric Johannsen	N/A
pENTR-BDLF2	Eric Johannsen	N/A
pENTR-BGLF1	Eric Johannsen	N/A
pENTR-BRRF2	Eric Johannsen	N/A
pENTR-BVRF1	Eric Johannsen	N/A
pENTR-BVRF2	Eric Johannsen	N/A
pENTR-BBRF1	Eric Johannsen	N/A
pENTR-B-GD-RF1	Eric Johannsen	N/A
pENTR-BXLF2	Eric Johannsen	N/A
pENTR-BALF4	Eric Johannsen	N/A
pENTR-gp350/220	Eric Johannsen	N/A
pENTR-BOLF1	Eric Johannsen	N/A
pENTR-BcLF1	Eric Johannsen	N/A
pENTR-BALF1	Eric Johannsen	N/A
pENTR-BHRF1	Eric Johannsen	N/A
pENTR-BARF1	Eric Johannsen	N/A
pENTR-BaRF1	Eric Johannsen	N/A
pENTR-BFRF2	Eric Johannsen	N/A
pENTR-BORF2	Eric Johannsen	N/A
pENTR-BPLF1 1–1000	Eric Johannsen	N/A
pENTR-BPLF1 501–1500	Eric Johannsen	N/A
pENTR-BPLF1 1001–2000	Eric Johannsen	N/A
pENTR-BPLF1 1501–2500	Eric Johannsen	N/A
pENTR-BPLF1 2001–3000	Eric Johannsen	N/A
pENTR-BPLF1 3001–3149	Eric Johannsen	N/A
pENTR-BNLF2a	Eric Johannsen	N/A
pENTR-BDLF4	Eric Johannsen	N/A
pDEST-CMV-N-EGFP BILF1	This study	N/A
pDEST-CMV-N-EGFP-BXLF1	This study	N/A
pDEST-CMV-N-EGFP-MAVS (wildtype)	This study	N/A
pDEST-CMV-N-EGFP-MAVS (K362R/K371R)	This study	N/A
pDEST-CMV-N-EGFP-MAVS (K461R)	This study	N/A
pLIX402-Zta	This study	N/A
pLIX402-BRLF1	This study	N/A
pLIX402-BDLF3.5	This study	N/A
pLIX402-BALF5	This study	N/A
pLIX402-BGLF3.5	This study	N/A
pLIX402-BNLF2b	This study	N/A
pLIX402-BKRF3	This study	N/A
pLIX402-BVLF1.5	This study	N/A
pLIX402-BLLF3	This study	N/A
pLIX402-BRRF1	This study	N/A
pLIX402-BFLF2	This study	N/A
pLIX402-BGLF3	This study	N/A
pLIX402-LF1	This study	N/A
pLIX402-SM	This study	N/A
pLIX402-BALF1/0	This study	N/A
pLIX402-BFRF1	This study	N/A
pLIX402-LF2	This study	N/A
pLIX402-BGLF4	This study	N/A
pLIX402-BGLF5	This study	N/A
pLIX402-BFLF1	This study	N/A
pLIX402-BXLF1	This study	N/A
pLIX402-BALF3	This study	N/A
pLIX402-BBLF2/3	This study	N/A
pLIX402-BcRF1	This study	N/A
pLIX402-BBLF4	This study	N/A
pLIX402-BSLF1	This study	N/A
pLIX402-BALF2	This study	N/A
pLIX402-BNRF1	This study	N/A
pLIX402-BBLF1	This study	N/A
pLIX402-BLRF1	This study	N/A
pLIX402-BFRF0.5	This study	N/A
pLIX402-BKRF2	This study	N/A
pLIX402-BLLF2	This study	N/A
pLIX402-BLRF2	This study	N/A
pLIX402-BCRF1	This study	N/A
pLIX402-BFRF3	This study	N/A
pLIX402-BKRF4	This study	N/A
pLIX402-BSRF1	This study	N/A
pLIX402-BZLF2	This study	N/A
pLIX402-BDLF3	This study	N/A
pLIX402-BILF2	This study	N/A
pLIX402-BXRF1	This study	N/A
pLIX402-BBRF2	This study	N/A
pLIX402-BDLF1	This study	N/A
pLIX402-BILF1	This study	N/A
pLIX402-BGLF2	This study	N/A
pLIX402-BdRF1	This study	N/A
pLIX402-BMRF2	This study	N/A
pLIX402-BORF1	This study	N/A
pLIX402-BTRF1	This study	N/A
pLIX402-BBRF3	This study	N/A
pLIX402-BDLF2	This study	N/A
pLIX402-BGLF1	This study	N/A
pLIX402-BRRF2	This study	N/A
pLIX402-BVRF1	This study	N/A
pLIX402-BVRF2	This study	N/A
pLIX402-BBRF1	This study	N/A
pLIX402-B-GD-RF1	This study	N/A
pLIX402-BXLF2	This study	N/A
pLIX402-BALF4	This study	N/A
pLIX402-gp350/220	This study	N/A
pLIX402-BOLF1	This study	N/A
pLIX402-BcLF1	This study	N/A
pLIX402-BHRF1	This study	N/A
pLIX402-BARF1	This study	N/A
pLIX402-BaRF1	This study	N/A
pLIX402-BFRF2	This study	N/A
pLIX402-BORF2	This study	N/A
pLIX402-BPLF1 1–1000	This study	N/A
pLIX402-BPLF1 501–1500	This study	N/A
pLIX402-BPLF1 1001–2000	This study	N/A
pLIX402-BPLF1 1501–2500	This study	N/A
pLIX402-BPLF1 2001–3000	This study	N/A
pLIX402-BPLF1 3001–3149	This study	N/A
pLIX402-BNLF2a	This study	N/A
pLIX402-BDLF4	This study	N/A
PHAGE-3X FLAG-HA MAVS	This study	N/A
PHAGE-3X FLAG-HA BILF1	This study	N/A
PHAGE-3X FLAG-HA BXLF1	This study	N/A
PHAGE-3X FLAG-HA UFM1	This study	N/A
pDEST-CMV-N-mCherry BXLF1	This study	N/A
pDEST-CMV-N-mCherry BILF1 (wildtype)	This study	N/A
pDEST-CMV-N-mCherry BILF1 (ΔN)	This study	N/A
pDEST-CMV-N-mCherry BILF1 (ΔC)	This study	N/A
pDEST-CMV-N-mCherry BILF1 (C174A)	This study	N/A
pDEST-CMV-N-mCherry BILF1 (K122A)	This study	N/A

Software and algorithms		

Database for Annotation, Visualization and Integrated Discovery (DAVID)	Laboratory of Human Retrovirology and Immunoinformatics	https://david.ncifcrf.gov/home.jsp
GraphPad Prism 7	GraphPad Software	https://www.graphpad.com/scientific-software/prism/
Flowjo X	Flowjo LLC.	https://www.flowjo.com/
Biorender	Biorender	https://biorender.com/
ImageJ	ImageJ	https://imagej.nih.gov/ij/
ImageJ- ComDet	ImageJ	https://imagej.net/imagej-wiki-static/Spots_colocalization_(ComDet)
Zeiss Zen Lite (Blue)	Zeiss	https://www.zeiss.com/microscopy/int/products/microscope-software/zen-lite.html
Arivis Vision4D	Arivis	https://imaging.arivis.com/en/imaging-science/arivis-vision4d
AlphaFold Collab	Highly accurate protein structure prediction with AlphaFold^73^	https://colab.research.google.com/github/deepmind/alphafold/blob/main/notebooks/AlphaFold.ipynb#scrollTo=pc5-mbsX9PZC
Robetta Server	Protein structure prediction service^114^	https://robetta.bakerlab.org/
The PyMOL Molecular Graphics System, Version 2.0	Schrödinger, LLC	http://www.pymol.org/pymol
R	R Core Team^116^	https://www.R-project.org/
R-shiny	*shiny: Web Application Framework for R.* R package version 1.7.2.9000^117^	https://shiny.rstudio.com/
PEAKS X Pro	Bioinformatics Solutions Inc.	https://www.bioinfor.com/peaks-xpro/

Other		

Standard Fetal Bovine Serum, Qualified, USDA-Approved Regions	Thermo Fisher Scientific	10437028
RPMI 1640 Medium	Life Technologies	11875085
DMEM, high glucose, pyruvate	Life Technologies	11995081
Ham’s F-12 Nutrient Mix, GlutaMAX^™^ Supplement	Thermo Fisher Scientific	31765035

## Highlights

- First EBV interactome identifies key EBV-host and EBV-EBV interactions in lytic B cells
- Common host protein nodes targeted by multiple human herpesviruses identified
- EBV BILF1 drives MAVS lysine 461 UFMylation to block NLRP3 inflammasome activation
- UFM1 and Parkin trigger selective degradation of mitochondrial membrane cargo
