# Supplementary material for: An Epstein-Barr virus protein interaction map reveals NLRP3 inflammasome evasion via MAVS UFMylation
Source: Mol Cell. Author manuscript; Available in PMC 2023 Jul 27. (PMC10372749; doi:10.1016/j.molcel.2023.05.018)
Supplement: Figures S1–S6 and supplemental reference [file NIHMS1912437-supplement-Figures_S1_S6_and_supplemental_reference.pdf]

Molecular Cell, Volume *83*

Supplemental information

An Epstein-Barr virus protein interaction map reveals NLRP3 inflammasome evasion via MAVS UFMylation

Stephanie Pei Tung Yiu, Cassie Zerbe, David Vanderwall, Edward L. Huttlin, Michael P. Weekes, and Benjamin E. Gewurz

Supplemental Figure and Table legends:

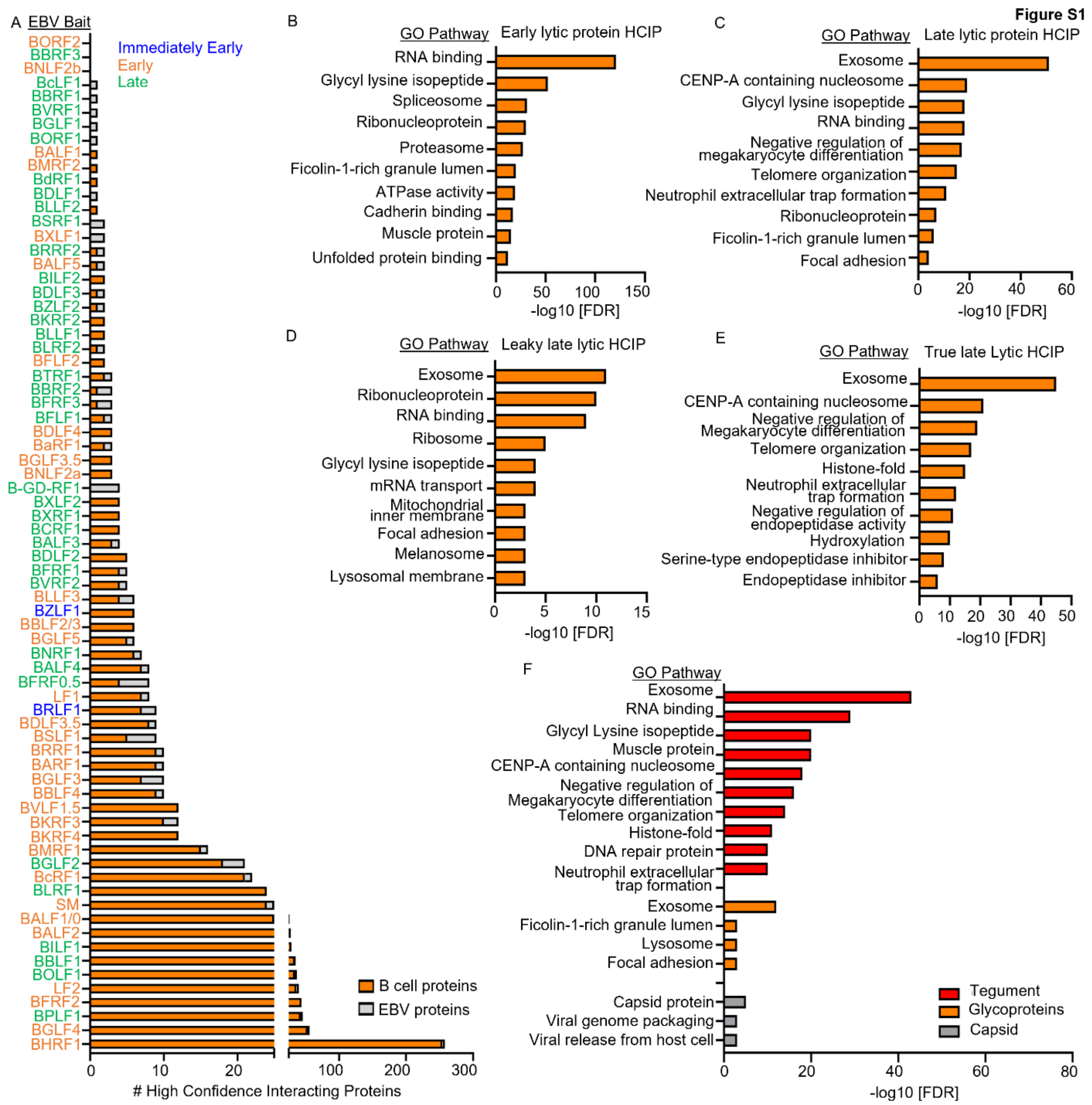

Figure S1. Related to Figures 1 : EBV-host protein-protein interaction network.

(A) Number of HCIPs per bait. Names of immediate early, early and late are color coded, as are the number of B-cell vs EBV protein interactors, as indicated.

(B-F) Bar graphs showing DAVID-derived enriched Gene Ontology (GO) pathways for host protein interactors of all EBV early (B); late (C); leaky late (D); true late lytic (E); and capsid,

tegument or glycoproteins (F), determined in this study. Leaky and true late lytic proteins were categorized according to the previous report[1].

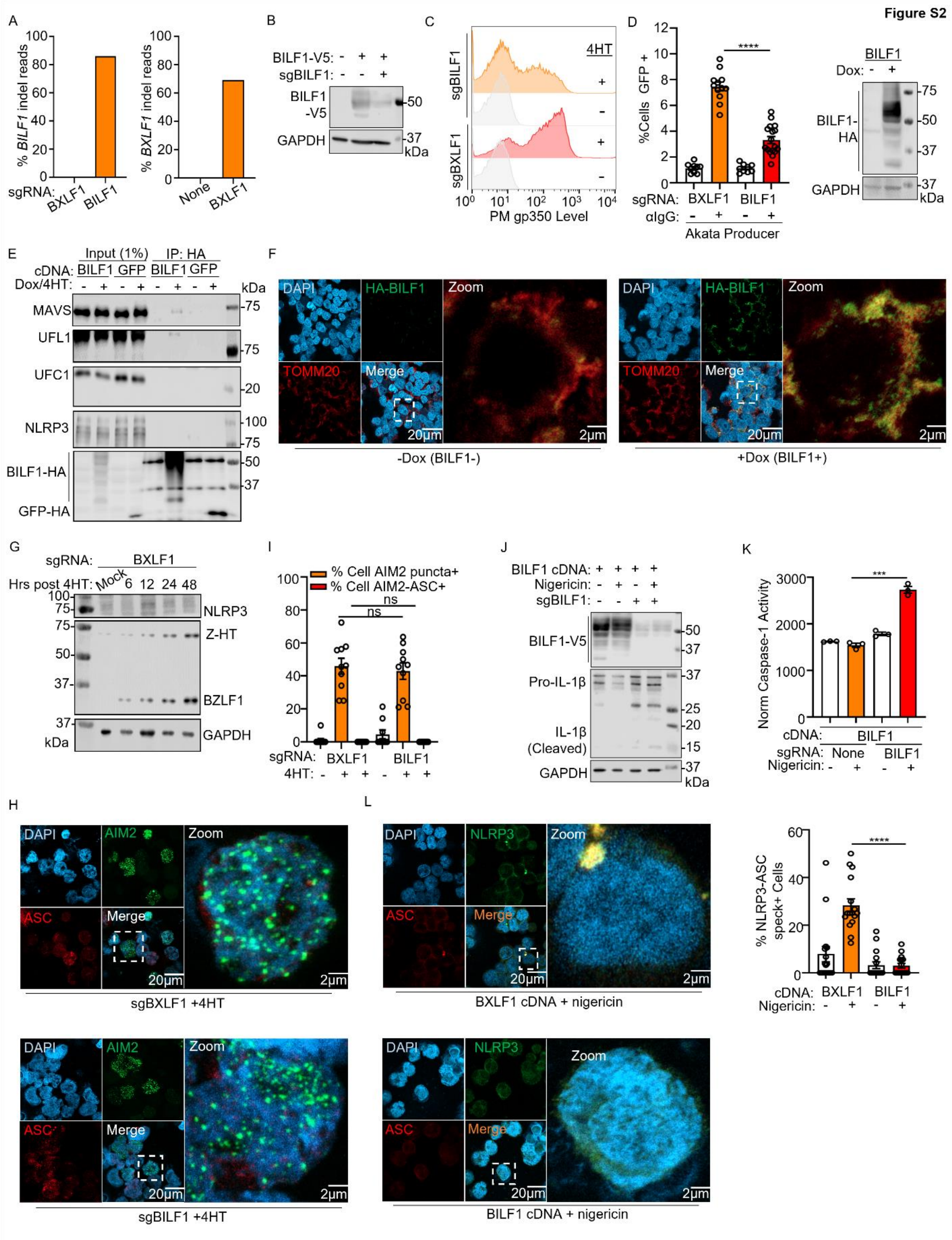
Figure S2
A
% BILF1 indel reads
sgRNA: BXLF1 BILF1
% BXLF1 indel reads
None BXLF1
B
BILF1-V5: - + +
sgBILF1: - - +
BILF1 -V5
GAPDH
kDa
C
sgBILF1
sgBXLF1
4HT
PM gp350 Level
D
%Cells GFP +
****
sgRNA: BXLF1 BILF1
αIgG: - + - +
Akata Producer
BILF1
Dox: - +
BILF1-HA
GAPDH
kDa
E
Input (1%) IP: HA
cDNA: BILF1 GFP BILF1 GFP
Dox/4HT:
kDa
MAVS
UFL1
UFC1
NLRP3
BILF1-HA
GFP-HA
F
DAPI
HA-BILF1
Zoom
TOMM20
Merge
20µm
2µm
-Dox (BILF1-)
+Dox (BILF1+)
G
sgRNA: BXLF1
Hrs post 4HT: Mock 6 12 24 48
NLRP3
Z-HT
BZLF1
GAPDH
kDa
I
% Cell AIM2 puncta+
% Cell AIM2-ASC+
ns
ns
sgRNA: BXLF1 BILF1
4HT: - + + - + +
J
BILF1 cDNA: + + + +
Nigericin: - + - +
sgBILF1: - - + +
BILF1-V5
Pro-IL-1β
IL-1β (Cleaved)
GAPDH
kDa
K
Norm Caspase-1 Activity
***
cDNA: BILF1
sgRNA: None BILF1
Nigericin: - + - +
H
AIM2
ASC
sgBXLF1 +4HT
sgBILF1 +4HT
L
NLRP3
BXLF1 cDNA + nigericin
BILF1 cDNA + nigericin
% NLRP3-ASC speck+ Cells
****
cDNA: BXLF1 BILF1
Nigericin: - + - +

Figure S2. Related to Figure 3: BILF1 is an inhibitor for NLRP3 inflammasome activation during EBV lytic cycle.

(A) Bar graphs showing the percentage of indel reads in *BILF1* (left) and *BXLF1* (right) genes in P3HR-1 cells expressing the indicated sgRNA.

(B) Immunoblot analysis of WCL from Akata Burkitt cells constitutively expressing BILF1-V5 cDNA and anti-BILF1 sgRNA, as indicated. Representative of n=2 replicates.

(C) FACS plot of PM gp350 levels in P3HR-1 cells expressing the indicated sgRNA mock-induced or induced into lytic cycle by 4HT for 24 h. Representative of n=3 replicates.

(D) Mean ± SEM from n=3 replicates of green Daudi assay analysis of infectious EBV titers from Akata Burkitt with BXLF1 or BILF1 sgRNA induced by IgG crosslinking for 48 h from n=3 replicates.

(E) Immunoblot analysis of 1% input and anti-HA immunopurified BILF1 or GFP complexes from P3HR-1 untreated or treated with doxycycline and 4HT for 24 h, as indicated. Representative of n=2 independent experiments. *indicates UFL1, beneath a non-specific band in the input.

(F) Immunofluorescence analysis of BILF1-HA mitochondrial localization, as determined by staining for HA and TOMM20 in P3HR-1 cells with or without the expression of BILF1 cDNA, induced by 5 μM doxycycline for 24 h.

(G) Immunoblot analysis of WCL from P3HR-1 cells expressing BXLF1 sgRNA, 4HT-induced for the indicated hours. Representative of n=2 replicates.

(H) Immunofluorescence analysis of formation of AIM2-ASC specks, as determined by staining for AIM2 and ASC in P3HR-1 cells expressing the indicated sgRNA induced into lytic cycle by 4HT for 24 h.

(I) Mean ± SEM percentage of cells with AIM2 puncta and AIMS-ASC specks, from n=3 replicates, as in (H), using data from 10 randomly selected panels of 120 nuclei, analyzed using ImageJ Comet plugin.

(J) Immunoblot analysis of WCL from Akata Burkitt cells constitutively expressing BILF1 cDNA and/or BILF1 sgRNA, treated with nigericin, as indicated, for 24 h. Representative of n=2 replicates.

(K) Mean ±SEM caspase-1 activity normalized by live cell number from n=3 replicates of Akata Burkitt cells expressing BILF1 cDNA and/or BILF1 sgRNA, treated with 10 μM nigericin for 4 h, as indicated.

(L) Immunofluorescence analysis of formation of NLRP3-ASC specks, as determined by staining for NLRP3 and ASC in Akata Burkitt cells with or without constitutively BILF1 cDNAs and treated with 10 μM nigericin for 4 h. Right: Mean ± SEM percentage of cells with NLRP3/ASC specks from n=3 replicates, as in the left panel, using data from 16 randomly selected panels of 300 nuclei, analyzed using ImageJ Comet plugin.

Statistical analysis was performed with Student T-test unless otherwise specified. ****p < 0.0001. ***p < 0.001. ns p > 0.05. White bars indicate scale.

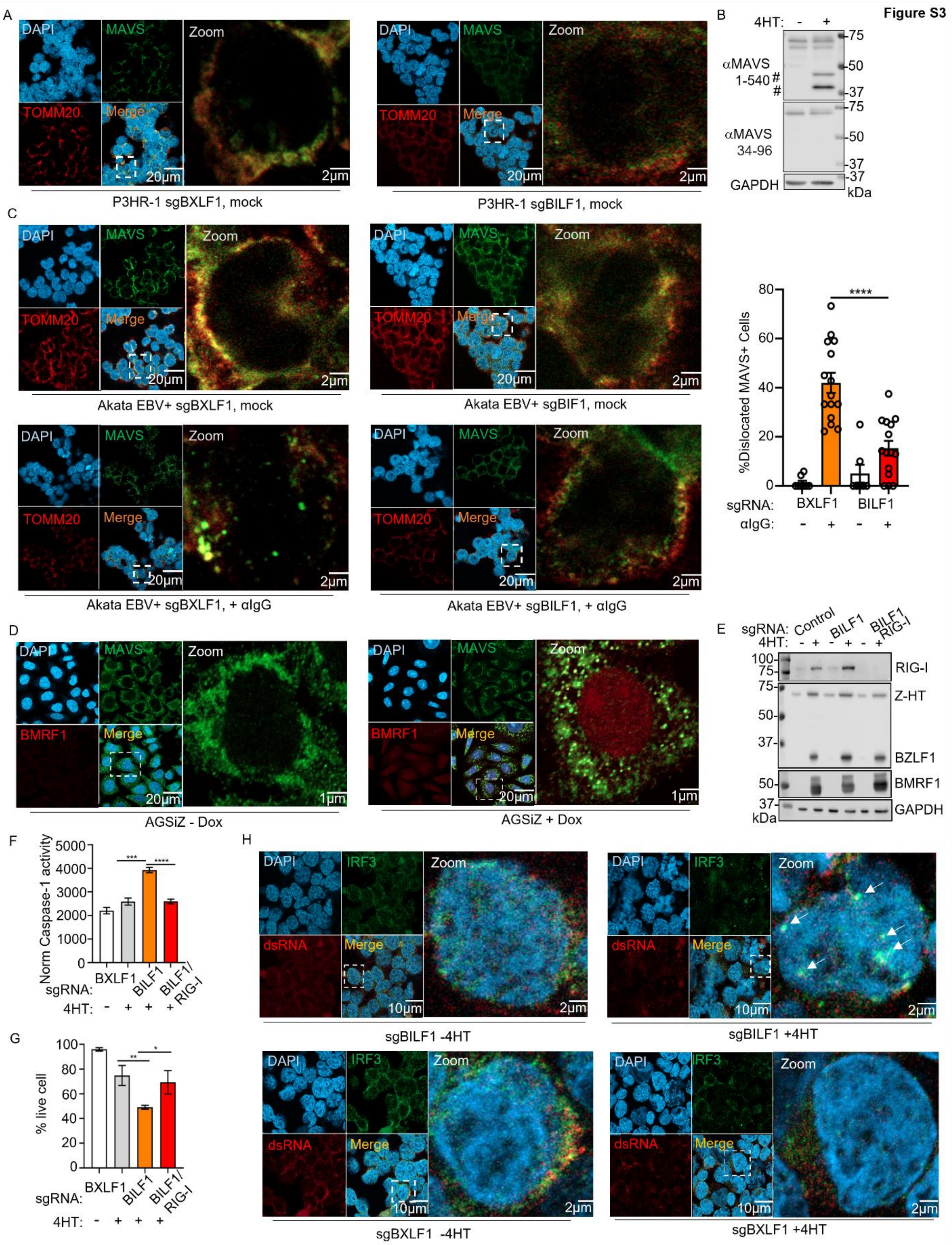
Figure S3
A
DAPI
MAVS
Zoom
TOMM20
Merge
20µm
2µm
P3HR-1 sgBXLF1, mock
P3HR-1 sgBILF1, mock
B
4HT: - +
αMAVS 1-540
#
#
αMAVS 34-96
GAPDH
75
50
37
kDa
C
Akata EBV+ sgBXLF1, mock
Akata EBV+ sgBIF1, mock
Akata EBV+ sgBXLF1, + αIgG
Akata EBV+ sgBILF1, + αIgG
%Dislocated MAVS+ Cells
****
sgRNA: BXLF1 BILF1
αIgG: - + - +
D
BMRF1
1µm
AGSiZ - Dox
AGSiZ + Dox
E
sgRNA: Control BILF1 BILF1 RIG-I
4HT: - + - + - +
RIG-I
Z-HT
BZLF1
BMRF1
GAPDH
F
Norm Caspase-1 activity
***
****
sgRNA: BXLF1 BILF1 BILF1/ RIG-I
4HT: - + + +
G
% live cell
**
*
H
IRF3
dsRNA
10µm
sgBILF1 -4HT
sgBILF1 +4HT
sgBXLF1 -4HT
sgBXLF1 +4HT

Figure S3. Related to Figure 4: BILF1 mediates MAVS sequestration that prevents MAVS-dependent NLRP3 recruitment to the mitochondria.

(A) Immunofluorescence analysis of MAVS mitochondrial dislocation, as determined by staining for MAVS and TOMM20 in mock-induced P3HR-1 cells expressing the indicated sgRNA.

(B) Immunoblot analysis of WCL from P3HR-1 cells mock induced or induced for lytic cycle by 4HT for 24 h, blotted with MAVS antibodies raised against the indicated peptide. # represents upper and lower molecular weight bands immunoreactive with anti-MAVS antibody.

(C) Immunofluorescence analysis of MAVS dislocation from the mitochondria, as determined by staining for MAVS and TOMM20 in Akata Burkitt cells expressing the indicated sgRNA mock-induced or induced into lytic cycle by IgG crosslinking for 48h. Right: Mean ±SEM percentage of cells with delocalized MAVS from n=3 replicates, as in the left panel, using data from 15 randomly selected panels of 300 nuclei, analyzed using ImageJ Comet plugin.

(D) Immunofluorescence analysis of MAVS dislocation in lytic-induced cells, as determined by staining for MAVS and BMRF1 in AGSiZ cells mock-induced or induced into lytic cycle by doxycycline for 24 h.

(E) Immunoblot analysis of WCL from P3HR-1 cells expressing the indicated sgRNA, treated with 4HT for 24 h, as shown. Representative of n=2 replicates.

(F) Mean ±SEM caspase-1 activity normalized by live cell number from n=3 replicates of P3HR-1 cells expressing the indicated sgRNA and treated with 4HT for 24 h, as shown.

(G) Mean ±SEM of live cell # from trypan blue stained P3HR-1 cells expressing the indicated sgRNA and induced by 4HT for 24 h.

(H) Immunofluorescence analysis of IRF3 subcellular distribution, as determined by cytosolic dsRNA and nuclear DAPI staining in sgBXLF1 (top) and sgBILF1 (bottom) cells mock-induced or induced into lytic cycle by 4HT for 24 h. White arrows represent IRF3 puncta in the nucleus.

Statistical analysis was performed with Student T-test unless otherwise specified. ****$p < 0.0001$. ***$p < 0.001$. **$p < 0.01$. *$p < 0.05$. White bars indicate scale.

Figure S4

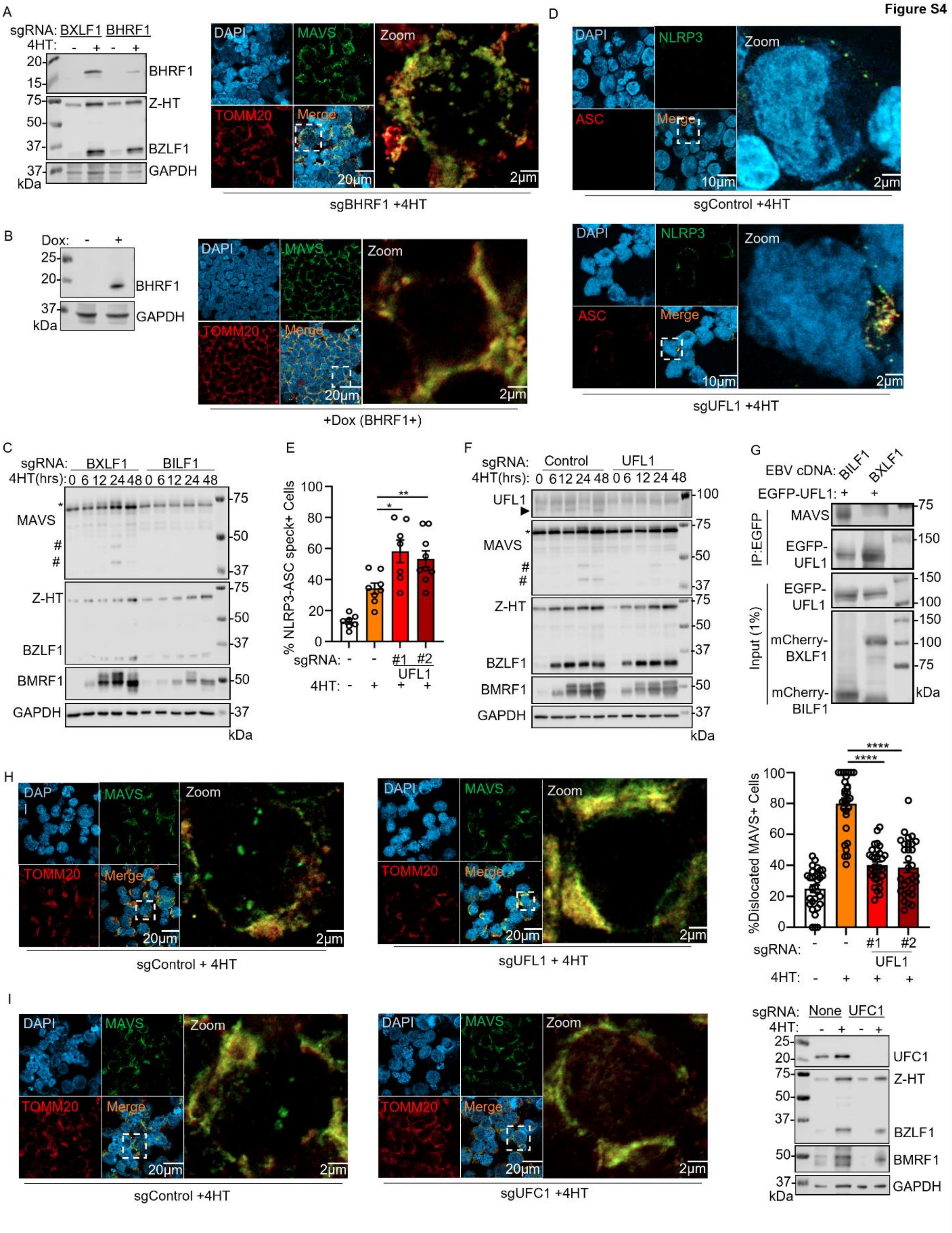
A
sgRNA: BXLF1 BHRF1
4HT: - + - +
BHRF1
Z-HT
BZLF1
GAPDH
kDa
DAPI
MAVS
TOMM20
Merge
Zoom
20μm
2μm
sgBHRF1 +4HT
B
Dox: - +
BHRF1
GAPDH
+Dox (BHRF1+)
C
sgRNA: BXLF1 BILF1
4HT(hrs): 0 6 12 24 48 0 6 12 24 48
MAVS
Z-HT
BZLF1
BMRF1
GAPDH
D
NLRP3
ASC
10μm
sgControl +4HT
sgUFL1 +4HT
E
% NLRP3-ASC speck+ Cells
sgRNA: - - #1 #2
UFL1
4HT: - + + +
F
sgRNA: Control UFL1
UFL1
G
EBV cDNA: BILF1 BXLF1
EGFP-UFL1: + +
IP:EGFP
EGFP-UFL1
Input (1%)
mCherry-BXLF1
mCherry-BILF1
H
sgControl + 4HT
sgUFL1 + 4HT
%Dislocated MAVS+ Cells
I
sgControl +4HT
sgUFC1 +4HT
sgRNA: None UFC1
UFC1

Figure S4. Related to Figures 5: BILF1 mediates MAVS UFMylation through UFL1.

(A) Left: Immunoblot analysis of WCL from P3HR-1 cells expressing the indicated sgRNA, treated with 4HT for 24 h, as indicated. Representative of n=2 replicates. Right: Immunofluorescence analysis of MAVS dislocation from mitochondria, as determined by staining MAVS and TOMM20 in P3HR-1 expressingh BHRF1 sgRNA and treated with 4HT for 24h.

(B) Left: Immunoblot analysis of WCL from P3HR-1, doxycycline induced for BHRF1 cDNA expression as indicated for 24 h. Representative of n=2 replicates. Right: Immunofluorescence analysis of MAVS dislocation from mitochondria, as determined by staining MAVS and TOMM20 in P3HR-1 cells expressing BHRF1 cDNA induced by doxycycline for 24 h, as indicated.

(C) Immunoblot analysis of WCL from P3HR-1 cells expressing the indicated sgRNA and mock induced or induced for lytic reactivation by 4HT for 6, 12, 24 and 48 h. High molecular weight versus low molecular weight bands reactive with anti-MAVS antibodies are denoted with * and #, respectively. Representative of n=3 replicates.

(D) Immunofluorescence analysis of NLRP3-ASC speck formation, as determined by staining for NLRP3 and ASC in P3HR-1 cells expressing control or UFL1 sgRNA and treated with 4HT for 24 h, as shown.

(E) Mean ± SEM percentage of cells with NLRP3/ASC specks from n=3 replicates, as in D, using data from 10 randomly selected panels of 200 nuclei, analyzed using ImageJ Comet plugin.

(F) Immunoblot analysis of WCL from P3HR-1 cells expressing the indicated sgRNA and mock induced or induced for lytic reactivation by 4HT for 24 h. UFL1 bands are denoted with a black arrow, respectively. High molecular weight versus low molecular weight bands reactive with anti-MAVS antibodies are denoted with * and #, respectively. Representative of n=3 replicates.

(G) Immunoblot analysis of 1% input and immunopurified EGFP-UFL1 complexes from 293T cells transfected with EGFP-UFL1 and BILF1 or BXLF1 cDNAs for 24h, as indicated. Representative of n=2 independent replicates.

(H) Left: Immunofluorescence analysis of MAVS dislocation from mitochondria, as determined by staining MAVS and TOMM20, in P3HR-1 cells expressing control or UFL1 sgRNA and stimulated by 4HT for 24 h, as indicated. Right: Mean ±SEM percentage of cells with dislocated MAVS from n=3 replicates, using data from 30 randomly selected panels of 300 nuclei, analyzed using ImageJ Comet plugin.

(I) Left: Immunofluorescence analysis of MAVS dislocation from mitochondria, as determined by MAVS and TOMM20 staining in P3HR-1 cells expressing control or UFC1 sgRNA, induced into lytic cycle by 4HT for 24 h. Right: Immunoblot analysis of WCL from P3HR-1 cells expressing the indicated sgRNA and treated with 4HT for 24 h, as indicated.

Statistical analysis was performed with Student T-test unless otherwise specified. ****$p < 0.0001$. **$p < 0.01$. *$p < 0.05$. White bars indicate scale.

Figure S5

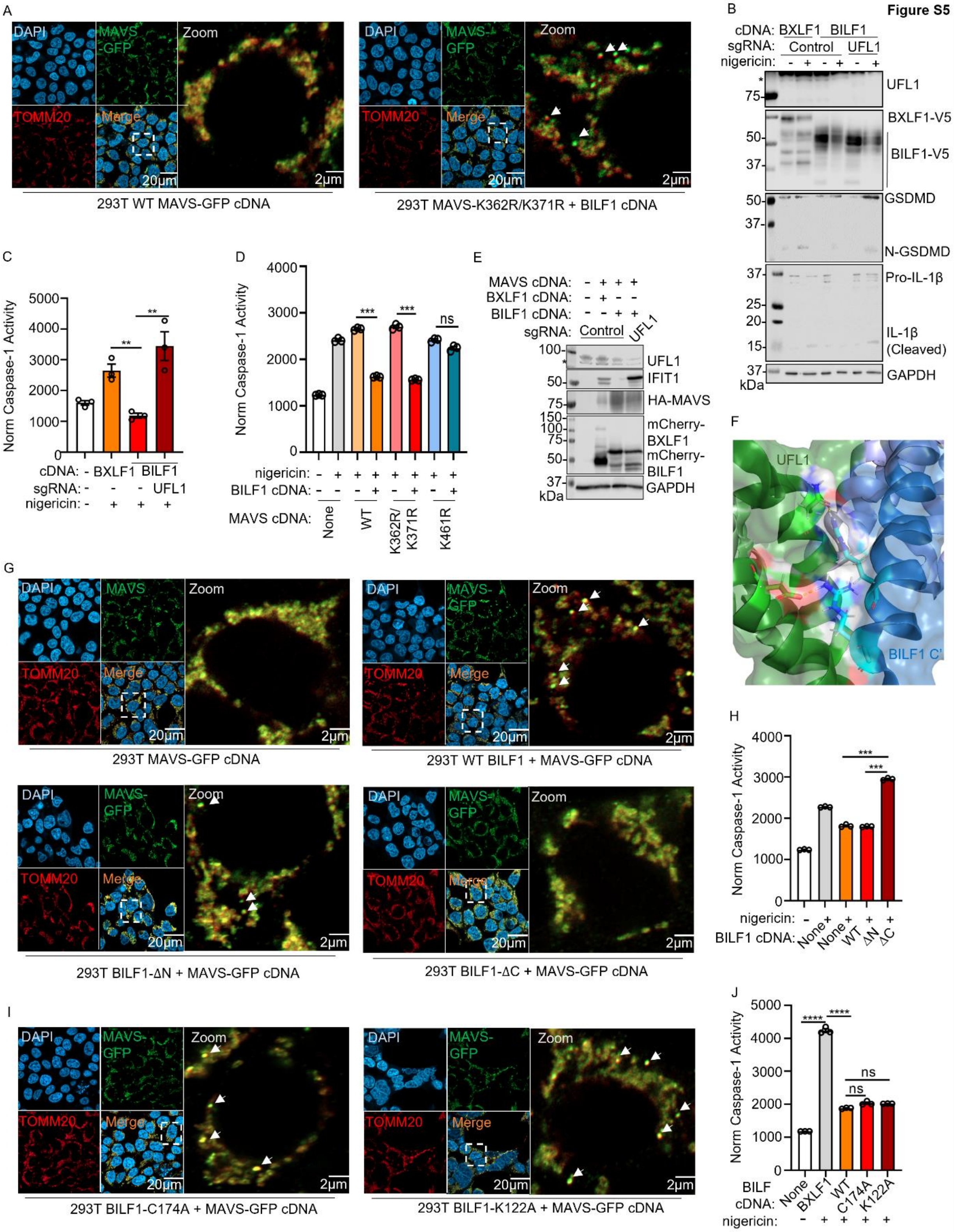
A
DAPI
MAVS-GFP
Zoom
TOMM20
Merge
20μm
2μm
293T WT MAVS-GFP cDNA
293T MAVS-K362R/K371R + BILF1 cDNA
B
cDNA: BXLF1 BILF1
sgRNA: Control UFL1
nigericin: - + - + - +
UFL1
BXLF1-V5
BILF1-V5
GSDMD
N-GSDMD
Pro-IL-1β
IL-1β (Cleaved)
GAPDH
kDa
C
Norm Caspase-1 Activity
cDNA: - BXLF1 BILF1
sgRNA: - - - UFL1
nigericin: - + + +
D
nigericin:
BILF1 cDNA:
MAVS cDNA: None WT K362R/K371R K461R
ns
E
MAVS cDNA:
BXLF1 cDNA:
BILF1 cDNA:
sgRNA: Control UFL1
UFL1
IFIT1
HA-MAVS
mCherry-BXLF1
mCherry-BILF1
GAPDH
kDa
F
UFL1
BILF1 C'
G
293T MAVS-GFP cDNA
293T WT BILF1 + MAVS-GFP cDNA
293T BILF1-ΔN + MAVS-GFP cDNA
293T BILF1-ΔC + MAVS-GFP cDNA
H
Norm Caspase-1 Activity
nigericin: - + + + +
BILF1 cDNA: None None WT ΔN ΔC
I
293T BILF1-C174A + MAVS-GFP cDNA
293T BILF1-K122A + MAVS-GFP cDNA
J
Norm Caspase-1 Activity
BILF cDNA: None BXLF1 WT C174A K122A
nigericin: - + + + +
ns

Figure S5. Related to Figure 5: MAVS is sequestered into mitochondrial-derived vesicles.

(A) Immunofluorescence analysis of WT vs K362R/K371R MAVS subcellular distribution, as determined by staining for GFP and TOMM20 in 293T cells co-transfected with cDNAs expressing BILF1 and GFP-tagged WT vs MAVS K362R/K371R for 24 h. White arrows indicate dislocated MAVS puncta.

(B) Immunoblot analysis of WCL from 293T cells expressing BILF1 or BXLF1 cDNA and control or UFL1 targeting sgRNA, treated with nigericin for 24 h, as indicated.

(C) Mean ±SEM caspase-1 activity normalized by live cell number from n=3 replicates as in (B).

(D) Mean ±SEM caspase-1 activity normalized by live cell number from n=3 replicates of 293T expressing the indicated BILF1 and MAVS cDNAs and treated with nigericin for 24 h, as shown.

(E) Immunoblot analysis of WCL from 293T cells expressing BILF1, BXLF1 or MAVS cDNA and control or UFL1 targeting sgRNA as indicated, for 24 h.

(F) Model of BILF1 and UFL1 interaction domains, as determined by Robetta software. Potential interacting residues are highlighted.

(G) Immunofluorescence analysis of MAVS dislocation from mitochondria, as determined by staining for GFP and TOMM20 in 293T cells transfected with wildtype, ΔN or ΔC BILF1 cDNA and MAVS-GFP cDNAs for 24 h, as indicated. White arrows indicate dislocated MAVS puncta.

(H) Mean ±SEM caspase-1 activity normalized by live cell number from n=3 replicates of 293T cells expressing the indicated cDNA and treated with nigericin for 24 h.

(I) Immunofluorescence analysis of MAVS dislocation from the mitochondria, as determined by staining for GFP and TOMM20 in 293T cells transfected with C174A or K122A BILF1 and MAVS-GFP cDNA for 24 h.

(J) Mean ±SEM caspase-1 activity normalized by live cell number from n=3 replicates of 293T cells expressing the indicated cDNA and treated with nigericin for 24 h, as indicated.

Statistical analysis was performed with Student T-test unless otherwise specified. ****$p < 0.0001$. ***$p < 0.001$. **$p < 0.01$. ns $p > 0.05$. White bars indicate scale.

Figure S6

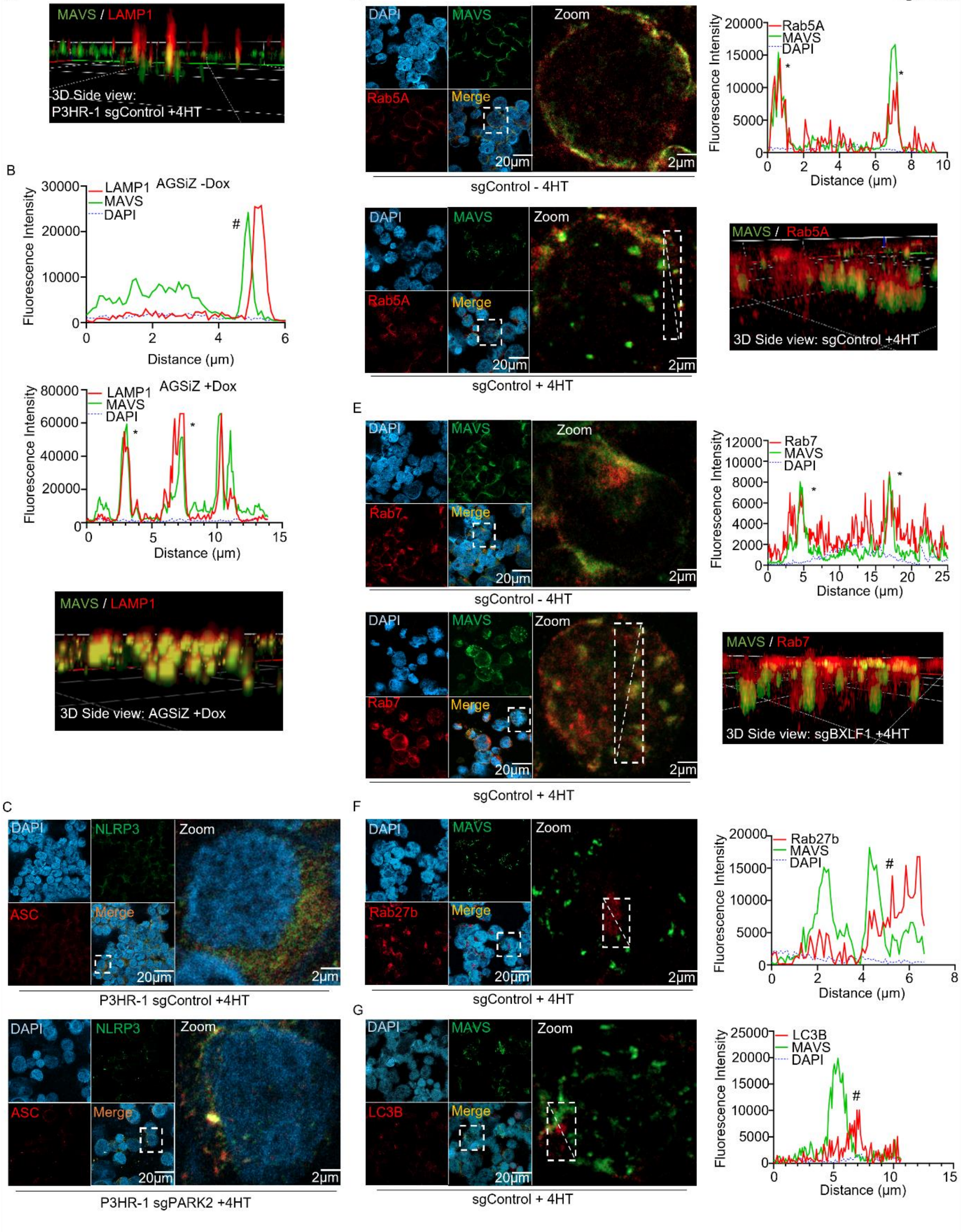
A
MAVS / LAMP1
3D Side view:
P3HR-1 sgControl +4HT
B
AGSiZ -Dox
LAMP1
MAVS
DAPI
Fluorescence Intensity
Distance (μm)
AGSiZ +Dox
3D Side view: AGSiZ +Dox
C
DAPI
NLRP3
ASC
Merge
Zoom
20μm
2μm
P3HR-1 sgControl +4HT
P3HR-1 sgPARK2 +4HT
D
Rab5A
MAVS
sgControl - 4HT
sgControl + 4HT
MAVS / Rab5A
3D Side view: sgControl +4HT
E
Rab7
3D Side view: sgBXLF1 +4HT
F
Rab27b
G
LC3B

Figure S6. Related to Figure 6-7: MAVS-containing mitochondrial-derived vesicles fuse with endosomes.

(A) 3D reconstruction of P3HR-1 cells with control sgRNA expression and 4HT induced for lytic replication stained with anti-MAVS (green) vs anti-LAMP1 (red) antibodies, as in Figure 6A. Representative of n=3 independent experiments.

(B) Top: Line scanning of fluorescence intensity for LAMP1 (red) and MAVS (green) in annotated the white rectangle in Figure 6C. # and * marks non-colocalization and colocalization, respectively. Bottom: 3D reconstruction of cells. Representative of three independent experiments.

(C) Immunofluorescence analysis of NLRP3-ASC specks formation, as determined by staining for NLRP3 and ASC in P3HR-1 cells expressing the indicated control or PARK2 targeting sgRNAs. Cells were 4HT induced for lytic replication for 24 h.

(D) Left: Immunofluorescence analysis of MAVS co-localization with early endosomes, as determined by staining for MAVS and Rab5A in P3HR-1 cells mock-induced or induced into lytic cycle by 4HT for 24 h. Right: Line scanning of fluorescence intensity for Rab5A and MAVS in the annotated white rectangle. * marks colocalization. 3D reconstruction of cells as in the bottom left panel. Representative of n=3 independent experiments.

(E) Left: Immunofluorescence analysis of MAVS co-localization with late endosomes, as determined by staining for MAVS and Rab7 in P3HR-1 cells mock-induced and induced into lytic cycle by 4HT for 24 h. Right: Line scanning of fluorescence intensity for Rab7 and MAVS in annotated the white rectangle. * marks colocalization. 3D reconstruction of cells as in the bottom left panel. Representative of three independent experiments.

(F) Left: Immunofluorescence analysis of MAVS colocalization with exosomes, as determined by staining for MAVS and Rab27b in P3HR-1 cells induced into lytic cycle by 4HT for 24 h. Right: Line scanning of fluorescence intensity for Rab27b and MAVS in the annotated white rectangle. # marks non-colocalization.

(G) Left: Immunofluorescence analysis of MAVS colocalization with autophagosomes, as determined by staining for MAVS and LC3B in P3HR-1 cells induced into lytic cycle by 4HT for 24 h. Right: Line scanning of fluorescence intensity for LC3B and MAVS in the annotated white rectangle. # marks non-colocalization. White bars indicate scale.

REFERENCES

1. Djavadian, R., Hayes, M., and Johannsen, E. (2018). CAGE-seq analysis of Epstein-Barr virus lytic gene transcription: 3 kinetic classes from 2 mechanisms. PLoS Pathog *14*, e1007114. 10.1371/journal.ppat.1007114.
